# It Runs in the Family: A Study of Political Candidacy Among Swedish Adoptees

**DOI:** 10.1007/s11109-017-9429-1

**Published:** 2017-09-20

**Authors:** Sven Oskarsson, Christopher T. Dawes, Karl-Oskar Lindgren

**Affiliations:** 1 Department of Government, Uppsala University and UCLS, Box 514, 751 20 Uppsala, Sweden; 2 Wilf Family Department of Politics, New York University, 19 W. 4th Street, 2nd Floor, New York, NY 10012 USA; 3 Department of Government, Uppsala University, IFAU, and UCLS, Box 514, 751 20 Uppsala, Sweden

**Keywords:** Political candidacy, Intergenerational transmission, Adoption study, Pre- and post-birth effects, Role modeling

## Abstract

What motivates citizens to run for office? Recent work has shown that early life parental socialization is strongly associated with a desire to run for office. However, parents not only shape their children’s political environment, they also pass along their genes to those same children. A growing area of research has shown that individual differences in a wide range of political behaviors and attitudes are linked to genetic differences. As a result, genetic factors may confound the observed political similarities among parents and their children. This study analyzes Swedish register data containing information on all nominated and elected candidates in the ten parliamentary, county council, and municipal elections from 1982 to 2014 for a large sample of adoptees and their adoptive and biological parents. By studying the similarity in political ambition within both adoptive and biological families, our research design allows us to disentangle so-called “pre-birth” factors, such as genes and pre-natal environment, and “post-birth” factors like parental socialization. We find that the likelihood of standing as a political candidate is twice as high if one’s parent has been a candidate. We also find that the effects of pre-birth and post-birth factors are approximately equal in size. In addition, we test a number of potential pre- and post-birth transmission mechanisms. First, disconfirming our expectations, the pre-birth effects do not seem to be mediated by cognitive ability or leadership skills. Second, consistent with a role modeling mechanism, we find evidence of a strong transmission in candidacy status between rearing mothers and their daughters.

**Electronic supplementary material:**

The online version of this article (doi:10.1007/s11109-017-9429-1) contains supplementary material, which is available to authorized users.

## Introduction

There are many recent examples of elected leaders that are scions of political dynasties. In the United States, the 43rd President, George W. Bush, is the son of the the 41st President, George H.W. Bush. George W. Bush’s opponent in the 2000 election, Albert Gore Jr., is the son of U.S. Senator Albert Gore. Mitt Romney, the Republican nominee for president in 2012, is the son of three term Michigan governor and 1968 presidential candidate George Romney. This phenomenon is not unique to the United States, with prominent examples in the Philippines (Benigno Aquino III, son of President Corazon Aquino), Malaysia (Najib Razak, son of Abdul Razak Hussein), Singapore (Lee Hsien Loong, son of Lee Kuan Yew), and Canada (Justin Trudeau, son of Pierre Trudeau).

Popular accounts of political dynasties suggest that being exposed to politics during their formative years strongly influenced the decision of children to follow in the footsteps of their parents and enter into politics. For example, Mitt Romney was influenced to run for office in part by his experience working for his father’s gubernatorial campaign as a young man as well as his father’s encouragement to enter into public service.^1^ George W. Bush documented how his father influenced his decision to go into politics in his book *41: A Portrait of My Father*.^2^

This anecdotal evidence suggests that there is a strong intergenerational link in political ambition. However, more comprehensive studies on the strength and nature of this link is scant. A number of studies have documented family ties in both the U.S. (Laband and Lentz 1985; Dal Bó et al. 2009; Gaddie 2003; Prewitt 1970) and other countries (Rossi 2016; Querubin 2016; Geys and Smith 2016) using information on candidates and legislators. This approach has yielded valuable insights into the distribution of political power and the persistence of political elites. But it also has some important shortcomings.

First, rather than just looking at those who run for or occupy an elected office, we should consider what distinguishes candidates from the rest of the population who are not candidates. This introduces severe data constraints on any prospective study on the intergenerational transmission of political candidacy. Running for elected office is a rare event in all democracies. For example, in a typical Swedish election—the empirical setting in this study—less than 1% of the electorate runs for office at the parliamentary, county council or municipal levels. Consequently, the joint occurrence of both a parent and his or her child running for office is exceedingly uncommon.

Second, the intergenerational incumbency effect that the dynastic literature attempts to identify provide only one of many potential explanations for the importance of family background in politics. Most importantly, parents not only shape their child’s environment, but also pass along their genes to those same children. A growing area of research has shown that individual differences in a wide range of political behaviors and attitudes are linked to genetic differences (Alford et al. 2005; Fowler et al. 2008; Dawes et al. 2014; Cesarini et al. 2014; Oskarsson et al. 2015; Bell et al. 2009; Bell and Kandler 2015; Cranmer and Dawes 2012; Dawes et al. 2015; Fazekas and Littvay 2015; Fowler and Dawes 2008, 2013; Dawes and Fowler 2009; Friesen and Ksiazkiewicz 2015; Hatemi et al. 2007, 2014; Klemmensen et al. 2012; Littvay et al. 2011; Loewen and Dawes 2012; Settle et al. 2009, 2010). As a result, any documented intergenerational association in the propensity to run for office may reflect a transmission of genetic endowments from parents to children, different life experiences, or a combination of both mechanisms.

This study analyzes Swedish register data containing information on all nominated and elected candidates in the ten parliamentary, county council, and municipal elections from 1982 to 2014 for two large samples: the first one consisting of all adoptees born between 1960 and 1980 and their adoptive and biological parents (N $\approx$ 10,000) and the second one including all non-adoptees from the same birth cohorts and their parents (N $\approx$ 2,000,000). The aim of the study is threefold. First, we estimate the overall transmission rate of political candidacy between parents and non-adopted children. Second, by studying the similarity in political ambition within both adoptive and biological families, our research design allows us to decompose this overall transmission estimate into so-called “pre-birth” factors, such as genes and pre-natal environment, and “post-birth” factors like parental socialization. Third, we will examine possible mechanisms driving the pre-birth and post-birth effects.

To preview our results, we find that the likelihood of standing as a political candidate more than doubles if one’s parent has been a candidate. Moreover, we demonstrate that the effects of post-birth factors, measured by adoptive parents’ tendency to run for office, and pre-birth factors, measured by biological parents’ tendency to run for office, are equal in size and approximately sum to the overall transmission rate. In addition, we test a number of potential pre-birth and post-birth transmission mechanisms. First, we use data from military conscription tests to show that neither cognitive ability nor personality traits related to leadership skills mediate the relationship between pre-birth factors and running for office. Second, we find that the post-birth transmission of political candidacy is direct in nature and not driven by intergenerational associations in education. Furthermore, consistent with a role modeling mechanism, we find evidence of a strong transmission of candidacy status between adoptive mothers and their daughters.

The rest of this paper is organized as follows: we begin with a brief review of the relevant literature, followed by a discussion of the empirical framework. We then explain the adoption process in Sweden, describe the construction of the dataset, and provide sample summary statistics. The next sections report the results from the basic models, numerous robustness checks, and explore possible pre-birth and post-birth transmission mechanisms. We conclude with a discussion of the main findings and their implications.

### Running for Office: The Importance of Family

Despite the lack of previous studies directly focusing on the intergenerational transmission in political candidacy, there are several strands of related literatures that are relevant for our purposes. Taken together, the arguments and evidence provided in these studies suggests two things: (i) we should expect a strong intergenerational association in the propensity to run for elected office; and (ii) we should expect this relationship to be mediated by both genetic and social factors.

Looking first at research on political candidates, the question of what motivates citizens to run for office has long been of interest to political science scholars. Nearly 70 years ago, Laswell (1948) identified the “political type” as “power seekers, searching out the power institutions of society [...] and devoting themselves to the capture and use of government” (p. 20). In Lasswell’s conception of the political type, an individual’s drive for power emanates from personal characteristics and motivations.^3^ In the tradition of Laswell, Fox and Lawless (2005) focus on personal attributes, such as attitudinal dispositions and life experiences, that underly an individual’s interest in becoming a candidate. As part of several studies, Fox and Lawless have identified a number of factors that influence what they refer to as “nascent ambition” (Fox and Lawless 2005, 2014; Lawless 2011). Among these factors, having politically active and encouraging parents stands out as one of the most important determinants of whether individuals have a desire to enter into politics.

Moreover, cognitive ability and personality traits, which are known to be heritable (Bouchard and McGue 2003), have been shown to predict political ambition. Fox and Lawless (2005) and Lawless (2011) report that having a competitive personality type is a significant predictor of considering a run for public office. Mirroring these results, two recent studies on gender differences in candidate emergence show that related personality traits such as election aversion (Kanthak and Woon 2015) and tolerance for interpersonal conflict (Schneider et al. 2016) influence individuals’ willingness to run for office. Using Swedish register data, Dal Bó et al. (2016) show that nominated and elected candidates score higher on cognitive ability than the population at large.

The aforementioned studies focus primarily on the supply-side of political candidates. A number of recent studies on political dynasties further suggest that demand-side factors could give rise to positive intergenerational associations in political candidacy. Above all, Dal Bó et al. (2009) find that political power is self-perpetuating in the sense that legislators who hold power for longer become more likely to have relatives entering Congress in the future. The underlying idea is that longer time in office will have downstream effects on the political success of relatives due to better connections with the local party organization and better name recognition among voters. This self-perpetuating effect has been replicated in Argentina (Rossi 2016) and the Phillippines (Querubin 2016). However, using very similar research designs, Fiva and Smith (2016) fail to find any evidence that incumbency has a causal effect on political dynasty formation in Norway and Van Coppenolle (2017) reports a zero-effect of tenure length on establishing or continuing a dynasty in the UK. The mixed evidence suggests that the demand-side mechanisms hypothesized to explain the formation of political dynasties may be of less importance in cases such as Sweden and Norway with party-centered and proportional systems (Geys and Smith 2016).

Turning next to mass political behavior, one robust empirical finding in previous research is a strong parent–child resemblance for a number of political traits. Among other things, several studies have found sizable intergenerational transmission rates in political participation and voter turnout (Beck and Jennings 1982; Plutzer 2002; Jennings and Niemi 2014). These findings have commonly been interpreted as reflecting the influence of parental socialization where some households cultivate political interest and knowledge which lays the groundwork for later participatory behavior among their children (Andolina et al. 2003; McIntosh et al. 2007; Jennings et al. 2009).

However, as acknowledged by socialization researchers, these studies have not been able to control for the possible influence of genetic endowments passed from parents to their children which may lead to upward bias in the effect of familial socialization (Stoker and Bass 2011). Substantiating these claims, a number of studies have found that genetic factors influence participatory acts and attitudes (Hatemi et al. 2007; Fowler et al. 2008; Dawes et al. 2014; Klemmensen et al. 2012; Fowler and Dawes 2008, 2013; Dawes et al. 2015). Of special relevance for our study is the results reported in Cesarini et al. (2014). Using an adoption sample matched to information on voter turnout in Sweden, Cesarini et al. (2014) show that both pre-birth and post-birth factors contribute in approximately equal amounts to the intergenerational transmission in turnout behavior.

A third strand of relevant literature concerns social mobility. There is a large empirical literature showing strong intergenerational associations in occupational status across many countries and time-periods (see e.g. Breen and Jonsson 2005 for an overview). Importantly, occupational traits similar to political ambition such as entrepreneurship and leadership have been demonstrated to be influenced by both pre-birth and post-birth factors (Shane et al. 2010; De Neve et al. 2013; Lindquist et al. 2015).

To summarize, previous research on candidate emergence, mass political participation and social mobility give us reason to expect a strong parent–child association in one’s inclination to run for office. Moreover, studies on these same traits also suggest that the intergenerational transmission in elite political participation may be explained by both social and genetic factors. Yet, we still have very limited knowledge regarding both the exact strength of the purported intergenerational correlation in candidacy and the relative importance of pre- and post-birth factors in accounting for this correlation. The purpose of the present study is to shed further light on these vital questions.

## Empirical Framework

The standard model of intergenerational transmission, estimated using data on children who were raised by their biological parents, is given by:

1 $$y^{oc}_{i}=\beta _0+\beta _1 y^{bp}_{i} +\epsilon ^{oc}_{i}$$

where $y_i^{oc}$ is an indicator variable that takes the value one if the individual ran for office and zero otherwise; subscript *i* indexes the family in which a child is born and raised; superscripts *oc* and *bp* denote the biological child and biological parent, respectively; and $\epsilon _i^{oc}$ is a child-specific disturbance. Following Björklund et al. (2006), we refer to such children as ownbirth children (hence the superscript *oc*). The term “children” is used to distinguish ownbirth children and adoptees from the parental generation. Without covariates in the regression, $\beta _1$ is simply the difference in the probability of running for office among children whose biological parent ran for office and those whose parent did not. Our analysis is based on elections held between 1982 and 2014 (when the children were between 2 and 54 years old) and our definition of running for office is standing as a candidate in at least one of these ten elections.

Under certain assumptions, described below, data on adoptees and their biological and adoptive parents can be used to decompose $\beta _1$ into two components—one measuring pre-birth factors ($\alpha _1$) and one measuring post-birth factors ($\alpha _2$). We model the transmission from the biological parent (*bp*) and the adoptive parent (*ap*) to the adopted child (*ac*) born in family *i* and adopted and reared in family *j* as:

2 $$y^{ac}_{j}=\alpha _0+\alpha _1 y^{bp}_{i} + \alpha _2 y^{ap}_{j} + \epsilon ^{ac}_{j}$$

Pre-birth factors include genetic endowments and, in case of maternal transmission, the quality of the uterine environment, whereas post-birth factors are all environmental effects that occur after birth. For children in family *i* who are not adopted, and thus are biologically related to their rearing parents, $y_i^{bp} = y_j^{ap}$. Therefore, Eq. 2 collapses to Eq. 1 with the implied restriction that $\alpha _1+\alpha _2=\beta _1$. In most adoption studies, $\alpha _1$ is indirectly inferred by estimating $\alpha _2$ in an adoption sample that only contains information on the adoptive (but not the biological) parents and $\beta _1$ in a sample of ownbirth children and their parents (Plug 2004; Sacerdote 2007). However, since the Swedish registries contain information on candidacy status for both the adoptive and the birth parents, our data allows us to estimate $\alpha _1$ and $\alpha _2$ directly and test the restriction that $\alpha _1+\alpha _2=\beta _1$.

As with all models, this framework is based on a number of assumptions. First of all, in order to interpret the $\alpha _1$ and $\alpha _2$ parameters as measures of pre- and post-birth factors, we need to make three assumptions. The first is that adoptees are randomly assigned to families or, alternatively, that the assignment process is related to observable factors we can control for, such as education, age or income. However, if authorities use unobserved information about the adoptees’ biological parents to try to find a set of adoptive parents with similar characteristics, the transmission coefficients obtained from the adoption sample are likely to be biased upward.

Second, since adoptees are never assigned to a family immediately after birth, it is also necessary to assume that variation in neo-natal environments is not a source of bias. Suppose, as is likely to be the case, that the neo-natal environment is positively correlated with the pre-birth environment. If the neo-natal environment impacts a later decision to run for office, the estimated pre-birth effect may be biased upward.

Third, it is crucial that the adoptees have no contact with, nor any information about, the biological parents at the time of the candidacy decision. For example, if an adoptee knows that his or her birth parent has run for office, then the estimated pre-birth effect on candidacy status will likely be biased upward.

To equate the sum of the population parameters $\alpha _1$ and $\alpha _2$ to $\beta _1$ we must make three additional assumptions. First, ownbirth and adopted children should not have systematically different pre-birth characteristics. Second, they should not face systematically different post-birth environments. More precisely, these two assumptions require that the three sets of parents—adoptive parents, biological parents to children given up for adoption, and parents with ownbirth children—are randomly drawn from the same distribution of parents. Third, adoptees should not be exposed to different environments, including parenting behaviors, just because they were adopted. For example, adopted children may not be as close to their parents as ownbirth children, on average, especially in those cases where there are striking differences in the physical appearance (Grotevant et al. 2000). Therefore, due to the fact that feelings of closeness between parent and child are positively related to successful transmission of political traits (Rico and Jennings 2015), extrapolations from adoption samples to samples of ownbirth children may be biased.^4^ In addition, the very process of adoption itself may be developmentally disruptive in ways that impact the transmission from parent to child.

As a first check of the plausibility of these identifying assumptions, we present an overview of the Swedish adoption system in the Online Appendix. The discussion focuses in particular on five key features of the system that are directly relevant for evaluating the plausibility of our identification strategy: (i) the timing of the child’s placement in the adoptive home; (ii) the formal rules and informal norms that determined the selection process used to match children to adoptive parents; (iii) the information about the biological parents available to the adoptees; (iv) how the adoptees compare to non-adopted children; and (v) how the adoptee’s biological parents compare to the adoptive parents.

Four important conclusions directly related to the identification assumptions discussed above follow from this description of the Swedish adoption system. First, in a large majority of cases the children were placed with their adoptive parents early after birth, most often between 3 and 12 months of age. Second, despite changes in the guidelines to social workers on matching children to parents with similar cognitive and physical characteristics, it seems likely that some selective placement persisted. Third, the adoptive parents in general had only limited information about the biological parents and according to evaluation studies only a small fraction of adult adoptees requested and obtained further information about their birth parents from the official records. Fourth, there are differences between the adoptive and the biological parents. Above all, adoptive parents tend to be somewhat older, better educated, and more likely to be employed in the white collar sector compared to the adoptees’ birth parents. However, as we will discuss in more detail later, these rather minor deviations from the ideal conditions do not seem to affect the substantive findings.

## Data and Summary Statistics

We construct our dataset using information from several administrative sources held by Statistics Sweden. Most importantly, Statistics Sweden maintains a comprehensive database called the Multi-Generation Registry (MGR). The register includes all individuals (so called index persons) born after 1931 who were also residents in Sweden at some point since 1961. The register contains information on the biological parents of individuals and, in those cases where an individual was adopted, the identity of the adoptive parents. For adoptees, however, the information on biological parents, especially fathers, is sometimes missing. The structure of the register makes it straightforward to identify parent–child pairs and many other first- and second-degree biological relationships for adoptees and non-adoptees alike.

We use the MGR to construct two samples: an adoption sample and an ownbirth sample. The MGR consist of 10,717,814 non-adopted individuals and 155,865 individuals adopted by at least one parent. We impose a number of restrictions on both samples.^5^ In our analyses we focus on children born between 1960 and 1980. The reason for this is twofold. First, until 1959, adoptions in Sweden were considered weak in the sense that some ties between the biological parents and the children they put up for adoption were not permanently cut. For instance, biological parents still had a responsibility to provide economic support to their children if needed and the adopted child had legal rights to receive inheritances from their biological parents (Bohman 1970; Nordlöf 2001). From 1959, all legal ties between the adopted children and their biological parents were permanently cut.

Second, the choice to restrict the samples to children born between 1960 and 1980 is guided by a trade-off between including children that are old enough and parents that are young enough to be considered as political candidates at some point during the ten consecutive elections between 1982 and 2014. In Table A2 in the Appendix, we report the baseline probabilities of running for office across the ten elections for adopted and ownbirth children born between 1960 and 1980 and their parents. Reflecting the fact that the children are young at the beginning of the study period whereas the parents are on average in their seventies by 2014 (see Table 1), the probability of standing as a candidate is increasing among children and decreasing among parents over time.

Furthermore, both the adoption and the ownbirth samples are restricted to children who were alive at the time of the first election for which they were eligible to vote and stand as a candidate. Likewise, we excluded all children both of whose rearing or biological parents had died by the time of the first election in 1982. We also used the quinquennial census records between 1960 and 1990 and yearly household information from 1991 and later to verify that the biological parents (for the ownbirth sample) or the adoptive parents (for the adoption sample) were the same persons listed as the household parents according to each census up to the age 16 (or the closest census year before turning 16).

Finally, the adoption sample is restricted to adoptees (i) for whom at least one biological parent is identified, (ii) who were adopted by two parents, and (iii) who were not adopted by any relative (such as aunts, uncles, and grandparents). After imposing these restrictions, the final samples consist of 1,958,341 non-adopted children (1,005,229 males and 953,112 females) and 10,141 adoptees (5,369 males and 4,772 females).

We matched all the individuals in the two samples to the Register of Nominated and Elected Candidates, which contains information on all nominated and elected candidates in the ten elections held between 1982 and 2014.^6^ We also matched children and parents to administrative registers with information regarding educational attainment, income, occupational status, and some additional demographic and socioeconomic characteristics.^7^

Table 1 reports summary statistics separately for the children and parents in the ownbirth (upper panel) and adoption (lower panel) samples. The first four columns provide information on the distribution of age, education, and occupational status. The entries show that adopted children (row 4) have slightly lower levels of education and are less likely to be employed in white collar jobs compared with the sample of ownbirth children (row 1). The adopted children are also almost four years older on average, reflecting the fact that the number of within-country adoptions decreased since 1970 (Nordlöf 2001).

Comparing the characteristics of ownbirth (rows 2 and 3) and adopted (rows 5 and 6) childrens’ biological parents, the differences are larger. Ownbirth childrens’ birth parents have about one more year of schooling and are more likely to occupy white-collar positions. Moreover, the biological parents of adoptees were two to three years younger on average when giving birth to their children.^8^ The fraction of teenage parents is also considerably higher among adopted children (27% of the mothers and 9% of the fathers) compared to ownbirth children (6% of the mothers and 1% of the fathers).

Comparing adoptive parents (rows 7 and 8) and ownbirth childrens’ biological parents (rows 2 and 3) reveals the opposite pattern, especially for the fathers. The adoptive parents are, on average, better educated, more often white-collar workers, and relatively older compared to the adopted childrens’ birth parents.

These differences substantiate the discussion of the Swedish adoption system in the Online Appendix. Adopted children appear to come from less advantaged families but are placed in families higher up on the socioeconomic ladder. However, despite these differences in means there is substantial overlap in the distributions of parental characteristics.

Lastly, descriptive statistics for our main outcome—an indicator for standing as a candidate at least once in the ten general elections held between 1982 and 2014—are provided in column 5. The sixth column displays the average number of candidacies among individuals running for office at least once during the study period. The baseline probability associated with running for office is about one percentage point higher among adopted children compared to ownbirth children. This is partly a reflection of the fact that the individuals in the former group are slightly older and more frequently have rearing parents who are politically active than the children in the latter group. When controlling for age and parent candidacy status, the difference in the baseline probability for running for office decreases to 0.5 percentage points.

As for the parents, the pattern is similar to the one found for education and occupational status. Adoptive parents are more likely to run for office compared to birth parents of adopted children. The baseline probabilities for parents in the ownbirth sample lie in between adoptive and birth parents in the adopted sample. There is also a clear gender difference in that fathers of adopted and non-adopted children are more likely to stand as candidates than the corresponding mothers.

Before proceeding to the result section, it should be noted that the bulk of the variation in our outcome measures is driven by candidates at the municipality level. More than 80% of the total number of nominees in our estimation sample pertains to individuals running at the municipal level. In view of this, it is important to note that Swedish municipalities play a crucial role in the provision of vital government goods and services such as social assistance and education. Much like the national parliament and county level assemblies, the municipal councils are elected using a party-list proportional system. The municipalities are governed by a “quasi-parliamentary system” where a majority party or coalition typically appoints committee leaders and determines local policy (Bäck 2003). Municipalities have independent income taxation rights and employ large shares of the labor force.^9^

**Table 1:** Summary statistics

	Age in 2014	Age at birth	Schooling	White-collar	Nominated (indicator)	Nominated (frequency)
*Ownbirth sample*						
(1) Children	44.6	–	12.6	57.7	2.27	1.93
	(5.9)	–	(2.2)	(49.4)	(14.9)	(1.44)
	[1,958,345]	–	[1,847,213]	[1,712,087]	[1,958,345]	[44,367]
(2) Fathers	75.0	30.4	10.5	51.2	5.11	2.99
	(9.3)	(6.4)	(3.0)	(50.0)	(22.0)	(2.20)
	[1,043,487]	[1,043,487]	[988,269]	[934,808]	[1,043,487]	[53,305]
(3) Mothers	72.0	27.4	10.4	32.4	3.34	2.74
	(8.7)	(5.6)	(2.8)	(46.8)	(18.0)	(2.04)
	[1,094,590]	[1,094,590]	[1,035,513]	[912,718]	[1,094,590]	[36,518]
*Adoption sample*						
(4) Children	48.2	–	12.0	48.6	3.33	1.94
	(4.8)	–	(2.0)	(50.0)	(18.0)	(1.58)
	[10,141]	–	[9,955]	[8,800]	[10,141]	[338]
(5) Fathers (biological)	75.8	27.9	9.3	32.1	2.74	2.17
	(9.1)	(8.0)	(2.5)	(46.7)	(16.3)	(1.51)
	[5,574]	[5,574]	[5,175]	[4,233]	[5,574]	[153]
(6) Mothers (biological)	71.9	23.6	9.5	20.9	2.48	2.26
	(7.7)	(6.2)	(2.4)	(40.6)	(15.6)	(1.84)
	[8,865]	[8,865]	[8,376]	[6,567]	[8,865]	[220]
(7) Fathers (adoptive)	83.4	35.2	10.6	58.8	6.83	2.91
	(7.9)	(5.5)	(3.2)	(49.2)	(25.2)	(1.98)
	[8,160]	[8,160]	[8,067]	[7,684]	[8,160]	[557]
(8) Mothers (adoptive)	80.9	32.6	10.1	35.1	3.79	2.80
	(7.6)	(5.1)	(2.9)	(47.7)	(19.1)	(1.93)
	[8,316]	[8,316]	[8,248]	[6,862]	[8,316]	[315]

Means, standard deviations (in parentheses), and number of observations (in brackets) for some key variables. Row 1 shows summary statistics for the non-adopted children in the sample; rows 2–3 display summary statistics for parents to the non-adopted children; row 4 shows summary statistics for the adopted children in the sample; rows 5–8 display summary statistics for parents to the adopted children. White-collar is the share (in percentage points) of individuals employed as legislators, senior officials, managers, professionals, technicians or associate professionals at some point between 1980–1990 (parents) or 2001–2010 (children). Nominated is the share (in percentage points) of individuals running for office at least once in the ten elections between 1982 and 2014. The last column displays the average number of candidacies among individuals running for office at least once during the ten elections between 1982 and 2014

## Results

### Baseline Results

Table 2 reports the intergenerational transmission coefficients for the sample of ownbirth (upper panel) and adoptive (lower panel) children. We use an indicator for being nominated at least once as our main outcome.^10^ We use linear probability models to facilitate comparisons with earlier adoption studies. However, as shown in the Online Appendix, the findings are substantively identical if we instead use a logit model. We report separate models for whether either parent (column 1), the father (columns 2 and 4), or the mother (columns 3 and 5) ran for office. In the sixth column we enter both paternal and maternal candidacy.

Throughout, we report estimates from models that include a set of baseline covariates. These covariates include the child’s gender, birth-year indicators for the child and each parent, and 24 county indicators of where each parent lived at the time of the child’s birth. Apart from these individual level controls, we also include the ratio of local council seats to the electorate in the municipality within which the child resides at the time of the election (*Seats-to-Voters*).^11^ As already noted, the variation in our outcome measures is mainly driven by candidates nominated at the municipality level. Moreover, local Swedish elections operate by a party-list system where local nomination committees largely control who gets nominated and how candidates are ranked on the list. Given that the number of candidates included on these party lists far from perfectly reflects the number of voters in a municipality, there will be a strong and mechanically negative relationship between the size of the electorate in the municipality and the chance of being nominated (Besley et al. 2013; Dancygier et al. 2015).

Consider, first, the intergenerational transmission of political candidacy in the ownbirth sample. The estimates in column 1 suggest that having a parent who ran for office is associated with an approximately 5 percentage points higher probability of standing as a candidate. Given a baseline probability of 2.27% among the ownbirth children (see Table 1), the magnitude of this transmission should be considered very large. According to columns 2 and 3, the candidate experience of mothers is somewhat more important. The likelihood of running for office is more than 6 percentage points higher for those having a politically active mother. The corresponding estimate associated with having a father run for office is 5.3 percentage points.

To further put the magnitude of the intergenerational transfer in perspective, Table 2 reports the effect of increasing the *Seats-to-Voters* ratio from the 10th to the 90th percentile. This corresponds to increasing the number of seats in a municipal assembly from one in every 1573 voters to one in every 209 voters. As expected, such a shift has a large impact on the probability of running for office. However, the size of this mechanical effect (2.4 percentage points) is less than half the size of the intergenerational transmission rate. Having politically active parents is thus a very important predictor of offspring candidacy status.

In columns 4–6 we turn to the potential bias stemming from parental concordance. Recent research has shown that the interspousal concordance in political attitudes is among the strongest of all social and biometric traits, including educational attainment, height and church attendance (Alford et al. 2011). Consistent with these findings, the tetrachoric correlation between mother’s and father’s candidacy status in the ownbirth sample is strong: r = 0.50,$p<0.01$.

The entries in column 6 display estimates from a model that includes both fathers’ and mothers’ candidacy experience simultaneously. For reasons of comparison, columns 4 and 5 report results from separate paternal and maternal models with the sample restriction that both mothers and fathers are non-missing. Reflecting the high degree of spousal concordance, the transmission coefficients from the separate paternal and maternal models reported in columns 4 and 5 (in the upper panel of Table 2) decrease in magnitude when including both parents’ candidacy status as covariates in the same model (column 6). The paternal effect goes from 0.053 to 0.045 and the maternal effect falls from 0.063 to 0.053. Thus, having both a mother and a father who ran for office is associated with an almost 10 percentage points higher probability of standing as a candidate.

The lower panel of Table 2 reports the results for the adoption sample. The estimates show that both pre-birth and post-birth factors are significant predictors of the adoptee’s likelihood of standing as a candidate. According to column 1, the pre-birth and post-birth effects are approximately equal in size. Holding the rearing parents’ candidacy status constant, the probability of running for office is 3.5 percentage points greater if one or both of the biological parents were nominated ($p<0.01$). Holding constant the biological parents’ candidacy experience, a child who had at least one rearing parent who ran for office is 3.4 percentage points more likely to stand as a candidate ($p<0.01$).

The estimates of father–child transmission in column 2 suggest that the pre-birth effect is almost twice as large as the post-birth effect. Strikingly, however, the mother–child association seems to be mainly due to a strong post-birth effect combined with a substantially weaker and statistically insignificant impact of the biological mother’s candidacy status. The sum of the biological and adoptive parent transmission coefficients is presented at the bottom of Table 2. Across the three specifications presented in columns 1, 2, and 3, the sum of the pre-birth and post-birth effects is slightly larger than the corresponding intergenerational association for ownbirth children in the upper panel. In no case, however, is the difference statistically significant.

Turning again to parental concordance, the interspousal correlations for candidacy status in the adoption sample are equal to 0.18 (biological mother–biological father, $p=0.012$) and 0.53 (adoptive mother–adoptive father, $p<0.01$). The fact that the correlation for the adoptive parents is much stronger than the corresponding correlation for the biological parents suggests that the upward bias in the paternal and maternal transmission coefficients in the upper panel in Table 2 is mainly driven by inflated post-birth effects. The results presented in columns 4–6 in the lower panel of Table 2, based on a sample restricted to children for whom we have information on both biological and adoptive mothers’ and fathers’ candidacy status, confirm this conjecture. Since many biological fathers are missing, the sample size declines significantly in comparison to the samples used in the separate paternal and maternal models (shown in columns 2 and 3). When taking spousal concordance in candidacy status into account (column 6), the post-birth effects decrease in size whereas the pre-birth effects are less affected.

Several important lessons can be drawn from these results. First, there is a strong parent–child transmission in the tendency to run for office. The baseline probability of standing as a candidate of 2.27% and the estimates of the transmission coefficient imply that having at least one parent who is or was a candidate is associated with a doubling of the likelihood of running for office.

Second, when considering the adopted children, we find evidence that the intergenerational transmission in candidacy status is composed of both pre-birth and post-birth factors. Having a biological parent who ran for office is a good predictor of the adoptee’s probability of running for office. This is despite the fact that all formal links between biological parents and children were broken at the time of adoption and a large majority of the children in the sample have no information about their biological parents (Nordlöf 2001).

Third, the estimates also indicate that adoptive parents’ political activity is a major source of intergenerational resemblance. All specifications show positive and significant transmission estimates for rearing fathers and mothers. Judging by the results based on the joint parental indicator in column 1 or the combined estimates in column 6 (of Table 2), approximately half of the intergenerational association in candidacy status is accounted for by pre-birth factors and half by post-birth factors.^12^

Fourth, the transmission from mothers to children seem to be somewhat stronger than the father–child association. Furthermore, whereas the paternal effect is driven by both pre-birth and post-birth factors, the link between mothers and their children is almost fully accounted for by the post-birth environment. Below, we argue that this pattern of results is consistent with a role modeling mechanism.

Finally, in order to avoid inflated transmission estimates due to the strong interspousal correlation in the tendency to run for office, it is important to include both mothers’ and fathers’ candidacy status jointly in the models.

In the Online Appendix we discuss and provide evidence of the internal and external validity of these results. In terms of internal validity, we show that (i) the small fraction of the adoptees that were placed with their adoptive parents after turning one year old are unlikely to bias the pre-birth and post-birth estimates to any great extent; (ii) the transmission estimates are not sensitive to non-random placement based on a large set of observable parental characteristics; (iii) restricting the sample to adoptees whose biological and adoptive parents lived in different counties does not alter the results; (iv) the transmission estimates are robust to using nominated but not elected parents; and v) reweighting the adoption sample such that the birth and adoptive parents are more similar to the parents in the ownbirth sample only marginally influences the transmission coefficients. As for external validity, we present transmission estimates for a number of political traits related to candidacy status in a Swedish and a US sample. These estimates are strikingly similar across the two countries.

**Table 2:** Transmission coefficients for political candidacy

	1	2	3	4	5	6
*Ownbirth sample*						
Parent	0.054***	–	–	–	–	–
	(0.001)	–	–	–	–	–
Father	–	0.053***	–	0.053***	–	0.045***
	–	(0.001)	–	(0.001)	–	(0.001)
Mother	–	–	0.063***	–	0.063***	0.053***
	–	–	(0.001)	–	(0.001)	(0.001)
Female	−0.004***	−0.004***	−0.004***	−0.004***	−0.004***	−0.004***
	(0.000)	(0.000)	(0.000)	(0.000)	(0.000)	(0.000)
Seats to voter	0.024***	0.025***	0.026***	0.025***	0.026***	0.024***
	(0.000)	(0.000)	(0.000)	(0.000)	(0.000)	(0.000)
*N*	1,694,804	1,635,951	1,674,697	1,591,302	1,591,302	1,591,302
*Adoption sample*						
Birth parent	0.035***	–	–	–	–	–
	(0.013)	–	–	–	–	–
Birth father	–	0.057**	–	0.052**	–	0.049**
	–	(0.023)	–	(0.024)	–	(0.024)
Birth mother	–	–	0.014	–	0.011	0.004
	–	–	(0.015)	–	(0.019)	(0.019)
Adoptive parent	0.034***	–	–	–	–	–
	(0.009)	–	–	–	–	–
Adoptive father	–	0.030**	–	0.035**	–	0.028**
	–	(0.013)	–	(0.014)	–	(0.014)
Adoptive mother	–	–	0.059***	–	0.041*	0.034
	–	–	(0.017)	–	(0.021)	(0.022)
Female	−0.007*	−0.009*	−0.011***	−0.013**	−0.014**	−0.015**
	(0.004)	(0.005)	(0.004)	(0.006)	(0.006)	(0.006)
Seats to voter	0.026***	0.022***	0.028***	0.021***	0.022***	0.020**
	(0.006)	(0.007)	(0.006)	(0.008)	(0.008)	(0.008)
Pre-birth + post-birth	0.069***	0.087***	0.074***	0.087***	0.052*	0.077***/0.038
	(0.016)	(0.026)	(0.022)	(0.028)	(0.028)	(0.027)/(0.028)
*N*	8,756	5,145	8,352	4,538	4,538	4,538

Linear regressions. In the upper panel the standard errors (in parentheses) are clustered by parent. In the lower panel the standard errors are clustered by adoptive parent. The columns display results using running for office at least once as outcome. All models include controls for childs gender, child birth-year dummies, parents birth-year dummies, and 24 dummies for parents’ county of residency. ***/**/*, indicates significance at the 1%/5%/10% level

### Pre- and Post-birth Mechanisms

Thus far we have presented results demonstrating that both pre-birth and post-birth factors predict candidacy status and that each account for approximately equally sized shares of the intergenerational association in the tendency to run for office. In the next step of the analysis, we investigate possible mechanisms that may mediate the pre- and post-birth effects. Turning first to the pre-birth effects, a common hypothesis is that any genetic effects on complex behavioral traits will be indirect and mediated by different psychological mechanisms such as cognitive ability and personality traits (Mondak et al. 2010). This hypothesis has been supported by previous research on mass political participation (Dawes et al. 2014, 2015).

To test whether cognitive ability and personality also mediate the pre-birth effects on elite political participation, we use two measures based on conscription data provided by the Military Archives of Sweden.^13^ Our measure of cognitive ability is based on the results from four subtests intended to capture logical, verbal, spatial, and technical abilities. To construct the indicator, we summed the scores of the four subtests and standardized the index such that the mean is equal to zero and the standard deviation is equal to one. The resulting measure has been shown to be a good measure of general intelligence (Carlstedt 2000) and positively predicts candidacy status in the Swedish context (Dal Bó et al. 2016).

Our measure of personality is based on the results from interviews with the conscripts conducted by psychologists. The main objective of the interview was to certify that the conscript could cope with the psychological requirements of the military service and, ultimately, war stress. Based on the interview, the psychologist ranked the conscript’s military aptitude along a nine-point Stanine scale with a mean of five and a standard deviation of two (Lindqvist and Vestman 2011). The measure we use in the analysis is a standardized version of this scale with mean zero and unit variance. It is important to note that this is a measure of a specific ability (military aptitude) rather than a specific trait. Military aptitude has been shown to tap into a bundle of different personality traits that we should expect to be positively related to candidacy status such as willingness to assume responsibility, emotional stability, independence, persistence, having an outgoing character and power of initiative (Fox and Lawless 2005; Lawless 2011; Kanthak and Woon 2015; Schneider et al. 2016). In addition, the measure of military aptitude used in this study has been reported to be highly correlated with leadership skills (Lindqvist and Vestman 2011).

We conduct a very simple test of the mediation hypothesis by comparing the estimated pre-birth effects when controlling and not controlling for cognitive ability and military aptitude. A significant decrease in the magnitude of the pre-birth effects would suggest mediation. Since conscription was mandatory only for males, the analysis is restricted to the male children in both samples. The results are presented in Table 3. To save space, we focus on models using the joint parental indicators.

As a benchmark, column 1 displays the transmission estimates from a model restricted to male children for whom we have information on cognitive ability and military aptitude. First, we can see that the transmission estimates from this restricted sample are very similar to the ones presented in column 1 of Table 2. Above all, the strong intergenerational transmission in candidacy status reflects both pre-birth and post-birth factors. Second, the results in column 2 suggest that cognitive ability is strongly related to candidacy status. A one standard deviation increase in cognitive ability is associated with a 0.6 percentage point increase in the probability of running for office in the ownbirth sample. The corresponding effect in the adoption sample is twice as large (0.013). Moreover, the coefficients for cognitive ability are weakly statistically different across the ownbirth and adoption samples ($p=0.09$).^14^ The effect of military aptitude is weaker and less precisely estimated, especially in the adoption sample. More importantly, controlling for cognitive ability and military aptitude does not alter the magnitude of the pre-birth effect. The estimated effect of having at least one parent who ran for office is 0.051 in both models. Consequently, the pre-birth transmission in candidacy status does not appear to be mediated by cognitive and non-cognitive skills as measured by the conscription tests.

Turning instead to the post-birth mechanisms, an important question concerns the extent to which the estimated post-birth effects reflect a causal link between political activity of the rearing parent and child. Such a pathway would operate whenever parents serve as role models that children emulate when creating their own political identities. Another possible mechanism explaining the link between parent and child political behavior is if parent activity levels shape the participation-relevant experiences and skills that their children acquire (Westholm 1999).

We can only indirectly and partly test these assertions with the data at our disposal. In a first step, we attempt to rule out the possibility that the parent–child link in candidacy status is driven by intergenerational associations in related attributes or traits. The most likely confounder in this instance is educational attainment. Based on previous research, we know that there is a significant association between education and the likelihood of running for office (Dancygier et al. 2015). Past studies have also reported a strong link between parental and child educational attainment (Björklund et al. 2006). Consequently, the estimated post-birth effects presented in Table 2 may reflect intergenerational transmission in education rather than a direct causal pathway between parent and child political activity.^15^ In a second step, we estimate separate models for sons and daughters in order to explore the extent to which the post-birth effects on candidate status reflect a role modeling mechanism.

The results of the first step are presented in columns 3 through 5 in Table 3. Column 5 reports estimates from a model controlling for parent and child years of schooling.^16^ The years of schooling variable has been recoded to the 0–1 range such that 0 denotes the sample minimum (7) and 1 the sample maximum (19). Columns 3 displays results from a corresponding baseline model with the sample restrictions implied by the inclusion of parental and child education, whereas column 4 presents estimates from a model including parental but not child education.

In the upper panel we can see that parental education is significantly related to the probability of standing as a candidate (model 4) and that this effect is almost entirely mediated by one’s own education (model 5). The pattern of results is similar in the adoption sample, although less precisely estimated. Comparing across models 3 and 5 it is also clear that the estimated transmission coefficients change only marginally, if at all, when controlling for parent and child years of schooling. Thus, the intergenerational transmission in candidacy status does not seem to be driven by the intergenerational association in educational attainment despite the fact that education itself is strongly related to candidacy status.

Next, we turn to the argument that the post-birth effects we observe may partly be explained by a role modeling mechanism. Recent research has shown that exposure to female role models promotes women’s political interest, ambition, and engagement. Atkeson (2003) reports an increased level of political engagement among women living in U.S. states with visible and competitive female candidates, whereas no such effect is detected among men in states with competitive all male races. Similarly, in two cross-national studies, Campbell and Wolbrecht (2006) and Wolbrecht and Campbell (2007) find that the presence of viable female political candidates and female members of parliament positively influences adolescent girls’ anticipated and adult women’s actual political involvement. Using Swiss data, Gilardi (2015) finds that the election of a woman in a given municipality increases the probability of female candidates in neighboring municipalities in the next election. On the other hand, Broockman (2014) fails to find any effect of a female candidate’s winning an election on other women running for office in U.S. state legislative elections.

Against this backdrop, we expect stronger transmission between mothers and daughters than between either mothers and sons or fathers and any child. Furthermore, the relative magnitudes of post-birth transmission rates across the different parent–child constellations, but not necessarily the pre-birth effects, should correspond to the pattern of transmission coefficients found in the ownbirth sample. To test this hypothesis, we allow the effects of politically engaged mothers and fathers to vary between daughters and sons.

Columns 6 and 7 in Table 3 display transmission estimates from such models. The results lend some support to the expectations. Above all, the post-birth transmission in candidacy status seem mostly to be driven by the influence of rearing mothers on their daughters. Looking first at the transmission estimates based on the ownbirth sample, the probability of standing as a candidate is 5.8 percentage points higher for daughters whose mothers ran for office. This effect is significantly stronger ($p<0.01$) than any of the other three transmission coefficients. Although less precisely estimated, the pattern of post-birth effects displayed in the lower panel is similar to the one obtained in the much larger ownbirth sample. The transmission from rearing parents to daughters appears to be predominantly due to the maternal influence ($p=0.051$). The adoptive mother–daughter effect is larger in magnitude than any of the other post-birth effects. However, in no case are these differences statistically significant. Finally, as was the case in the baseline models, the estimated pre-birth effects are clearly related to the sex of the parent. The influence of having a birth father who ran for office is positive whereas the maternal pre-birth effects are small in magnitude and insignificant. It should be noted, though, that the paternal pre-birth effects are somewhat stronger than (although not statistically different from) the corresponding maternal pre-birth effects.

**Table 3:** Pre- and post-birth mechanisms

	All	All	All	All	All	Sons	Daughters
*Ownbirth sample*							
Parent cand	0.056***	0.054***	0.054***	0.053***	0.052***	–	–
	(0.001)	(0.001)	(0.001)	(0.001)	(0.001)	–	–
Father cand	–	–	–	–	–	0.050***	0.037***
	–	–	–	–	–	(0.001)	(0.001)
Mother cand	–	–	–	–	–	0.045***	0.058***
	–	–	–	–	–	(0.002)	(0.002)
Cognitive ability	–	0.006***	–	–	–	–	–
	–	(0.000)	–	–	–	–	–
Military aptitude	–	0.001***	–	–	–	–	–
	–	(0.000)	–	–	–	–	–
Child years	–	–	–	–	0.038***	–	–
	–	–	–	–	(0.001)	–	–
Parent years	–	–	–	0.010***	0.001**	–	–
	–	–	–	(0.001)	(0.001)	–	–
*N*	742,115	742,115	1,675,397	1,675,397	1,675,397	806,948	764,538
*Adoption sample*							
Birth parent cand	0.051**	0.051**	0.037***	0.037***	0.036***	–	–
	(0.022)	(0.022)	(0.013)	(0.013)	(0.013)	–	–
Birth father cand	–	–	–	–	–	0.065*	0.033
	–	–	–	–	–	(0.038)	(0.031)
Birth mother cand	–	–	–	–	–	0.010	−0.006
	–	–	–	–	–	(0.034)	(0.020)
Adoptive parent cand	0.034**	0.032**	0.034***	0.033***	0.033***	–	–
	(0.014)	(0.014)	(0.009)	(0.009)	(0.009)	–	–
Adoptive father cand	–	–	–	–	–	0.030	0.027
	–	–	–	–	–	(0.021)	(0.020)
Adoptive mother cand	–	–	–	–	–	0.026	0.064*
	–	–	–	–	–	(0.029)	(0.033)
Cognitive ability	–	0.013***	–	–	–	–	–
	–	(0.004)	–	–	–	–	–
Military aptitude	–	0.001	–	–	–	–	–
	–	(0.003)	–	–	–	–	–
Child years	–	–	–	–	0.068***	–	–
	–	–	–	–	(0.013)	–	–
Adoptive parent years	–	–	–	0.004	−0.004	–	–
	–	–	–	(0.008)	(0.008)	–	–
*N*	3,801	3,801	8,662	8,662	8,662	2,357	2,118

Linear regressions. In the upper panel the standard errors (in parentheses) are clustered by parent. In the lower panel the standard errors are clustered by adoptive parent. The columns display results using running for office at least once as outcome. All models include controls for child gender, child birth-year dummies, parents birth-year dummies, and 24 dummies for parents’ county of residency. ***/**/*, indicates significance at the 1%/5%/10% level

## Conclusion

Family socialization has been shown to be one of the strongest predictors of nascent political ambition (Fox and Lawless 2005; Lawless 2011; Fox and Lawless 2014). However, as Stoker and Bass (2011, p. 463) caution, “building a better understanding of the pre-adult origins of political orientations and their subsequent development over the life span requires that genetic and biological traits be considered alongside mechanisms of social learning and influence.” In order to avoid genetic and environmental confounding when exploring the determinants of political ambition, it is necessary to utilize a research design that can disentangle these two factors. To achieve this, we analyze national, county council, and municipal election nominations for a large sample of Swedish adoptees and their adoptive and biological parents.

We find strong evidence in favor of intergenerational transmission of political ambition. The probability of being a candidate is 5 percentage points higher for individuals with politically active parents. Given the baseline probability of standing as a candidate of 2.27%, our transmission estimate implies that the likelihood of becoming a political candidate more than doubled if one’s parent had been a candidate.

Utilizing an adoption design, this transmission estimate can be decomposed into pre- and post-birth effects. We find that the effect of post-birth factors, such as parental socialization, is of equal size as pre-birth factors, which are comprised of genes and pre-natal environment. While our finding that post-birth factors are an important determinant of whether a child runs for office accords with previous research (Fox and Lawless 2005; Lawless 2011; Fox and Lawless 2014), our results suggesting that pre-birth factors are equally important underscore the point that omitting the genetic factors may inflate the influence of parental socialization.

As a follow up to this analysis, we sought to identify possible determinants of the pre-and post-birth transmission. We hypothesized that cognitive ability and personality traits related to leadership skills may mediate the transmission in candidacy status between birth parents and their children. Our findings did not support these expectations. Regarding the post-birth effects, our findings suggested that the intergenerational transmission in candidacy status was not confounded by intergenerational transmission in educational attainment but instead was more direct in nature. Moreover, based on a number of recent studies, we hypothesized and found some empirical support for the notion that maternal role modeling would lead to stronger transmission between rearing mothers and their daughters.

As we have pointed out, there are caveats that should be considered when evaluating our results. The adoption design requires a number of assumptions and violations of these assumptions may lead to bias in our estimates. However, based on extensive analyses probing the sensitivity of our results to potential violations, we believe it unlikely that the results are due to bias or confounding. With that said, we cannot directly validate every assumption so caution should nonetheless still be exercised when evaluating the findings. Our analysis is also based on one case, Sweden, and thus there are natural questions of external validity. While we have documented comparable rates of transmission within Sweden and U.S. samples for political behaviors similar to running for office, ideally our primary analysis will be replicated in other national contexts in the future. Finally, future research should also strive to enhance our understanding of the mechanisms underpinning the documented pre- and post-birth transmission effects. Our efforts to do so in this study were restricted by the lack of appropriate data. Whereas the data used in this study is well suited to provide very precise estimates of the transmission in candidacy status and to decompose this intergenerational association into pre- and post-birth factors, it has some limitations when it comes to pinpointing relevant causal mechanisms driving the pre- and post-birth effects.

In spite of these shortcomings, we believe that our study makes important contributions to research on political behavior in general and elite political participation in particular. First, to the best of our knowledge, ours is the first study to examine population wide data on parent–child pairs matched to information on all political candidates. Moreover, we do so over a 32-year period, covering ten elections, which permits us to provide very precise estimates of the intergenerational association in the propensity to run for office.

Second, by decomposing this association into pre-birth and post-birth effects, we go beyond the mere descriptive evidence of a strong transmission in candidacy status. In doing so, we add new knowledge to the twin study based research on individual differences in political participation by showing that not only mass participation (Hatemi et al. 2007; Fowler et al. 2008; Dawes et al. 2014; Klemmensen et al. 2012; Fowler and Dawes 2008, 2013; Dawes et al. 2015), but also elite political behavior is a partly genetically influenced trait.

However, a direct comparison of our findings with these twin studies is not possible, as we approach the question of nature and nurture from a different perspective. Twin studies are designed to decompose the total variation in a trait into unique environmental, shared environmental and genetic factors. An adoption design, as used in this study, serves to identify the variation in a trait that is due to intergenerational transmission. Given these differences, it is interesting to note that the results reported in existing twin studies on mass political participation align with a well-known general finding from a very large number of previous twin studies: the fact that shared environment accounts for preciously little, if any of the variation in just about any complex behavioral trait (Polderman et al. 2015). This empirical regularity has even been suggested as one of three “laws” of behavior genetics (Turkheimer 2000).^17^ This stands in stark contrast to the findings we present here. In particular, it is hard to reconcile the strong post-birth effects reported in this study with the absence of shared environmental influences in previous twin studies, especially since the set of post-birth mechanisms as understood in our study, such as role modeling, comprises a subset of all possible factors included under the umbrella of shared environment.

Instead, our results are more in line with findings of both pre- and post-birth effects reported in a number of previous adoption studies of different social, political and economic traits. Closely related to our study is the study by Cesarini et al. (2014) on the transmission in voter turnout in the 2010 Swedish general election. The authors of that study show that pre-birth and post-birth factors contribute approximately equally to produce the overall transmission in turnout behavior. However, apart from focusing on a different political trait, our study goes beyond Cesarini et al. (2014) in that we make an attempt to examine possible pre- and post-birth mechanisms and, in the latter case, are also able to find some support for a role modeling channel underlying the intergenerational association in political candidacy. The finding that pre- and post-birth effects are similar in size has also been reported for other traits such as education (Björklund et al. 2006) and criminal behavior (Hjalmarsson and Lindquist 2013). However, Lindquist et al. (2015) show that post-birth factors account for twice as much as pre-birth factors in the decomposition of the intergenerational association in entrepreneurship, a trait that at face value has much in common with political candidacy.

The differing conclusions based on twin and adoption studies speak to the need for multiple approaches when studying political and social behavior. Since twin and adoption studies have their own methodological strengths and weaknesses, and rest on different identifying assumptions, we need both methods to further our understanding of how nature and nurture interact to influence behavioral outcomes. Moreover, our study highlights the importance of correctly interpreting genetic and other pre-birth effects on political and social traits. Our finding that pre-birth factors are a strong predictor of political candidacy does not mean that there is direct causal link between a set of genetic factors and an individual’s propensity to run for office. Nor does it signal genetic determinism. Any genetic or other pre-birth effect on a complex behavior such as running for office will undoubtedly be mediated by a large set of factors, some of which are malleable (Jencks 1980).

As a hypothetical example, consider how small differences among children in partly inherited predispositions such as cognitive and non-cognitive skills may trigger divergent responses from parents, peers and teachers. In the long run, such processes may amplify initially small differences into large amounts of variation in traits that may influence adult political ambition. The fact that our investigation of possible mechanisms fails to find support for such pathways between pre-birth factors and candidacy status does not invalidate the argument as such. Instead, our inability to pinpoint the relevant pre-birth mechanisms should alert us to the need for further theorizing and more precise measurement. Such theoretical and empirical improvements are crucial for reaching a better understanding of how political behavior is transmitted across generations and may, eventually, provide important clues for policy initiatives aimed at reducing political inequalities.

## Electronic supplementary material

Below is the link to the electronic supplementary material.

Supplementary material 1 (pdf 251 KB)

## Footnotes

1
http://articles.latimes.com/2012/feb/25/nation/la-na-romney-michigan-20120226.

2 Chapter 5 of Lawless (2011) offers several additional examples.

3 For example, Laswell (1948) asserts that “power is expected to overcome low estimates of the self, by changing either the traits of the self or the environment in which it functions” (p. 39).

4 Although especially salient in the case of international or transracial adoptions, such mechanisms may also change the strength of the transmission process within ethnically homogenous samples such as ours.

5 See Table A1 in the Online Appendix for a detailed description of these sample restrictions.

6 In Sweden, the national and the two regional (county- and municipal-level) elections are held simultaneously on the third Sunday in September every three (until 1994) or four (after 1994) years. For the elections held in 1982, 1985, and 1988 we only have access to information on nominated and elected individuals at the municipality level. However, as explained below, a large majority of the total number of nominees in each election is comprised of candidates running in the municipality elections.

7 See the Online Appendix for additional details on these registers and variables.

8 Since the adopted children on average are somewhat older than the ownbirth children, the birth parents of adopted and ownbirth children are of similar age in 2014 (as shown in column 1).

9 In 2015, the average municipal income tax rate was approximately 21% and about 15% of the employed worked in the municipal sector.

10 In Online Appendix Tables A4 through A10, we also provide transmission coefficients from models based on several alternative outcomes: being nominated at least twice during the study period, the number of times parents and children ran for office during the study period, running for different levels of political office, winning office and being placed on the top half of the party list.

11 Each child is appointed the highest *Seats-to-Voters* value across the six elections held between 1991 and 2010.

12 The joint birth mother/father effect according to the estimates in column 6 is 0.053* ($p=0.068$). The corresponding adoptive mother and father effect is equal to 0.062** ($p=0.012$).

13 All men in our sample were required by law to participate in military conscription around the age of 18. For the men born after 1950 the military data has been digitalized. We were able to successfully match about 95% of the male children in our samples to the information in the Military Archives. For more detailed information about the tests taken at conscription and the resulting measures of cognitive and non-cognitive abilities, see the Online Appendix.

14 Additional analyses suggest that the effect of cognitive ability is positively related to child age in both samples. Since the average age is somewhat higher in the adoption sample, the interaction between cognitive ability and child age accounts for part of the differing ability effects on candidacy choices depending on one’s adoption status. At the same time, however, these results leave open the question of why the relationship between cognitive ability and candidacy status is related to age. Although interesting, we must leave this issue to future research.

15 The estimates for the intergenerational transmission in educational attainment (years of schooling) in our samples are very similar to the ones reported in Björklund et al. (2006). Specifically, when using the joint parental indicator for years of schooling, the transmission rate in the ownbirth sample is equal to 0.249 ($p<0.01$) whereas the pre- and post-birth effects amount to 0.107 ($p<0.01$) and 0.119 ($p<0.01$), respectively.

16 The years of schooling indicators were imputed based on the three-digit Swedish standard classification of education used in the the Swedish registers. For more details on this procedure, see the Online Appendix.

17 The three “laws” are: (i) all human behavioral traits are heritable; (ii) the influence of shared environment, such as being raised in the same family, is smaller than the influence of genes; and (iii) a large share of the variation in complex human traits is accounted for by unique environmental factors. On the basis of recent molecular studies, Chabris et al. (2015) propose a fourth “law”: complex human behavioral traits are influenced by many genetic variants, each of which accounts for a tiny amount of variation.
