# Supplementary material for: It Runs in the Family: A Study of Political Candidacy Among Swedish Adoptees
Source: Polit Behav. 2017 Sep 20;40(4):883–908. doi: 10.1007/s11109-017-9429-1 (PMC6514823; doi:10.1007/s11109-017-9429-1)
Supplement: Supplementary file 1 — Supplementary material 1 (pdf 251 KB) [file 11109_2017_9429_MOESM1_ESM.pdf]

Online Appendix to “It Runs in the Family. A Study of Political Candidacy among Swedish Adoptees”

Appendix A: Details on Data and Measures

This section provides a description of the data availability, data sources and variables used for the paper “It Runs in the Family? A Study of Political Candidacy among Adoptees”.

Data Availability

In this paper we use individual level information obtained from various administrative registers. The data are stored on an encrypted server at Statistics Sweden and all our analysis have been conducted through a remote desktop application. We are under contractual obligation not to disseminate these data to other individuals.

For interested researchers there are, however, two ways to get access to the administrative data used in this paper for replication purposes. The first possibility is to order the data directly from Statistics Sweden. Currently, Statistics Sweden require that researchers obtain a permission from a Swedish Ethical Review Board before data can be ordered (a description, in Swedish, of how to order data from Statistics Sweden is available at: `http://www.scb.se/sv_/Vara-tjanster/Bestalla-mikrodata`). We will also make available a complete list of the variables that we ordered from Statistics Sweden for this project.

The second possibility to replicate our analyses for interested researchers is to come

to Uppsala and reanalyze these data through the same remote server system that we used for our analyses. Any researcher interested in using this option needs to contact us before coming to Sweden so that we can arrange with Statistics Sweden that the researcher is temporarily added to our research team, which is required in order to get access to the remote server system.

Variables and Data Sources – Swedish Data

Nominated — Equal to 1 if the individual ran for office at the national, county or municipal level at least once in the ten general elections held 1982, 1985, 1988, 1991, 1994, 1998, 2002, 2006, 2010, and 2014. Information on the seven elections between 1991 and 2014 is retrieved from the Register of Nominated and Elected Candidates held at Statistics Sweden. For the elections in 1982, 1985, and 1988 the candidate information only covers the municipal level and is also available at Statistics Sweden.

Sex — Equal to 1 if male. Information is retrieved from the Swedish Population Register.

Birth Year — Information is retrieved from the Swedish Population Register.

County of Residence — The parents' county of residence according to the census closest after the child's birth (24 categories). Information is retrieved from the quinquennial censuses 1960 to 1990.

Seats-to-Voters — The ratio of the number of municipality council seats to the number of eligible voters in the municipality within which the child resides at the time of each of the six elections between 1991 and 2010. The data is retrieved from the Swedish Election Authority (www.val.se).

Years of Schooling — Educational attainment according to the three-digit Swedish standard classification of education (SUN 2000). Following the manual for classifying educational programmes in OECD countries (ISCED-97), we assigned the following years of schooling to each category: (old) primary school (7); (new) compulsory school (9);

(old) junior secondary education (9.5); high school (10-12 depending on the program); short university (13); longer university (14-16 depending on the program); short post-graduate (17); long post-graduate (19). The information on educational attainment is retrieved from the census in 1970 and the 1990, 2000 and 2010 waves of the Longitudinal integration database for health insurance and labour market studies (LISA by Swedish acronym).

Occupational Status — A dummy indicating whether the individuals were employed in the white collar sector at some point between 1980 and 1990 (parents) or 2001 and 2010 (children). The parental variable is based on the following occupational classification according to the censuses in 1980 and 1990: unskilled employees in goods production (1); unskilled employees in service production (2); skilled employees in goods production (3); skilled employees in service production (4); assistant non-manual employees, lower level (5); assistant non-manual employees, higher level (6); intermediate non-manual employees (7); other higher non-manual employees (8); upper-level executives (9); professionals (10); farmers (11); self-employed (excluding self-employed professionals and farmers) (12); non-classifed employees (13); old-age pensioners (14); housewives (or male equivalents) (15); students (16); and part-time workers (17). Categories 7-10 and 12 are defined as white collar occupations. The child variable is based on the three-digit Swedish standard classification of occupations (Ssyk96) according to the 2001-2010 waves of the Longitudinal integration database for health insurance and labour market studies. Categories 100-399 (corresponding to legislators, senior officials, managers, professionals, associate professionals and technicians) are defined as white collar occupations.

Turnout — Equal to 1 if the individual voted in one of the six Swedish parliamentary election between 1991 and 2010. The information is retrieved from the participation surveys carried out by Statistics Sweden in connection to each general election. The sample size in these surveys is around 50,000 to 100,000 individuals which approximately corresponds to 1-1,5% of the Swedish electorate.

Political Interest — Four-category indicator measuring whether the respondent engages in political discussions. This measure is based on the following survey item:

> How do you behave when you are in a group and political questions are discussed?
>
> (1) I don't listen when people talk politics
>
> (2) I usually listen, but I never participate in the discussion
>
> (3) I sometimes express my opinions
>
> (4) I usually participate in the discussion and voice my opinions

The information is retrieved from the annual Living Conditions Surveys 1980-2010 (ULF by Swedish acronym). The sample size in these surveys is around 10,000 individuals and the response average rate approximately 65-70%.

Attended Political Meeting — Dummy indicator measuring whether the respondent has attended a party meeting or political rally. This measure is based on the following survey item:

> Did you attend a meeting or rally organized by a political party (including youth and women's leagues) during the last 12 months?
>
> (1) Yes
>
> (2) No

The information is retrieved from the annual Living Conditions Surveys 1980-2010 (ULF by Swedish acronym).

Variables and Data Sources – U.S. Data

To obtain the U.S. transmission estimates presented in Table 4 in the main text we have used the "Three Generations Combined, 1965-1997 (ICPSR 4532)" study (TGC). Principal investigators for this study is Patrick Elliot (University of North Carolina,

Chapel Hill). This dataset combines three existing datasets: "Student-Parent Socialization Study, 1965 (ICPSR 07286)" (principal investigator: Kent Jennings (University of California, Santa Barbara)); "Youth-Parent Socialization Panel Study, 1965–1997: Four Waves Combined (ICPSR 04037)" (pricipal investigators: Kent Jennings (University of California-Santa Barbara), Gregory Markus (University of Michigan), Richard Niemi (University of Rochester), and Laura Stoker (University of California-Berkeley)); "Study of Political Socialization: Parent-Child Pairs Based on Survey of Youth Panel and Their Offspring, 1997 (ICPSR 04024)" (principal investigators: Kent Jennings (University of California-Santa Barbara) and Laura Stoker (University of California-Berkeley)). The data is downloadable from http://www.icpsr.umich.edu/icpsrweb/DSDR/studies/04532.

TGC is a four-wave panel study covering three biologically related generations of Americans. The original study was based on a national probability sample of 1,669 individuals who were high school seniors in 1965. We refer to these respondents as the original cohort, their parents as the parental cohort, and the children of the original cohort members as the third-generation cohort. The members of the original cohort have been surveyed four times: in 1965, 1973, 1982, and 1997. The 1997 survey attempted to include all third-generation cohort members who had reached an age of 15 or greater. Of the 1,669 original cohort members, 915 responded to the 1997 survey. Of these 915 individuals, 478 were parents of a total of 769 third-generation children who also responded to the survey. We restrict the sample to families that have no foster children living in them.

The following variables from the TGC dataset are used in the analyses:

Turnout (Parents) — Equal to 1 if the individual reported voting in the 1996 presidential election. The variable is constructed from responses to the question:

> In talking with people about the 1996 presidential election between Clinton, Dole, and Perot, we found that a lot of people weren't able to vote because they weren't registered or they were sick or they just didn't have time. How about you, did you vote or did something keep you from voting?

(1) Voted

(2) Did not vote

Turnout (Children) — Equal to 1 if the individual reported voting in the 1996 presidential election. The variable is constructed from responses to the question:

> Did you vote for President in 1996?
>
> (1) Voted
>
> (2) Did not vote
>
> (3) Too young to vote

Political Interest (Parents) — Four-category indicator measuring whether the respondent engages in politics. This measure is based on the following survey item:

> Some people seem to think about what's going on in government most of the time whether there's an election going on or not. Others aren't that interested. Would you say you follow what's going on in government most of the time, some of the time, only now and then, or hardly at all?
>
> (1) Most of the time
>
> (2) Some of the time
>
> (3) Only now and then
>
> (4) Hardly at all

Political Interest (Children) — Four-category indicator measuring whether the respondent engages in politics. This measure is based on the following survey item:

> Some people seem to think about what's going on in government most of the time, whether there's an election going on or not. Others aren't that interested. How often do you follow what's going on in government?
>
> (1) Most of the time
>
> (2) Some of the time

(3) Only now and then

(4) Hardly at all

Attended Political Meeting (Parents) — Dummy indicator measuring whether the respondent has attended a party meeting or political rally. This measure is based on the following survey item:

> Have you gone to any political meetings, rallies, dinners, or other things like that since 1982?
>
> (1) Yes
>
> (2) No

Attended Political Meeting (Children) — Dummy indicator measuring whether the respondent has attended a party meeting or political rally. This measure is based on the following survey item:

> Since 1982, have you gone to any political meetings, rallies, dinners, or other things like that?
>
> (1) Yes
>
> (2) No

Region of Residence (Parents) — Parental region of residence in 1973. Nine categories: New England (1); Middle Atlantic (2); East North Central (3); West North Central (4); Solid South (5); Border States (6); Mountain States (7); Pacific Coast (8); External States and Territories (9).

Race (Children) — Dummy indicators for the following three categories: Black (1); Hispanic (2); Other (3).

Sex (Children) — Equal to 1 if male.

Birth Year

Appendix B: Adoptions in Sweden 1960 to 1980

In this section we present a brief overview of the Swedish adoption system. Since our estimation sample is based on children born between 1960 and 1980, we will concentrate on the rules and norms regulating native born adoptions in Sweden during this time period. Our discussion focuses in particular on four key features of the system that are directly relevant for evaluating the plausibility of our identification strategy: (i) the timing of the child's placement in the adoptive home; (ii) the formal rules and informal norms that determined the selection process used to match children to adoptive parents; (iii) how the adoptees compare to non-adopted children; and (iv) how the adoptee's biological parents compare to the adoptive parents.[1]

The general guiding principle behind the Swedish adoption system was that the adoption should be conducted in a manner that is in the best interest of the child. An adopted child had the right to the same legal status as a biological child and at adoption, all formal connections with the biological parents were permanently cut. Adoptions were administered by a government representative and approved by a court. The identity of the biological mother and, whenever possible, the biological father of the adoptee were registered in the court documents.

The social worker's primary responsibility was to assess the prospective adoptive parents' ability to care for and raise the child. The typical adoption case involved an unmarried pregnant woman who did not have the means to provide for the child and therefore resorted to adoption. The first contact with the social authorities were often during pregnancy. The instructions of the social workers prescribed that the child ought to be separated from the biological mother as soon as possible after birth (Allmänna Barnhuset, 1955, 1969).

Before arriving to the home of the prospective adoptive parents, the newborn child

[1]This section is mainly based on the handbooks for social workers dealing with adoptions (Allmänna Barnhuset, 1955, 1969) and the evaluations of the adoption system provided in Bohman (1970) and Nordlöf (2001). Comprehensive discussions of these questions are also provided in Björklund et al. (2006), Hjalmarsson and Lindquist (2013), and Cesarini et al. (2014).

would first be placed in a special nursery. During this period, the biological mother was given some time to think over her decision and the child also underwent a comprehensive assessment of mental and physical health. The rules mandated that the child should not remain in the special nursery for more than three months and be placed in an adopted family on a trial basis before the age of six months. After the trial period, which lasted between three to six months, the prospective parents could formally apply for adoption. The court process was quite slow and it was not uncommon for the child to reach the age of two before the adoption was formally approved. By then, however, the child had usually lived in the adoptive home for over a year. Evaluation studies show that the prescribed recommendations on early placement in special nurseries and then adoptive families were followed in practice (Bohman, 1970; Nordlöf, 2001). Between 1960 and 1973, 86 percent of the children were placed in a special nursery before coming to their adoptive family. During the same time period, 83 percent of the babies arrived at their adoptive home before the age of one.

The official guidelines for social workers from 1955 explicitly instructed social workers to try to match children to parents with similar cognitive and physical characteristics (Allmänna Barnhuset, 1955). A decade and a half later, these recommendations were removed from an updated version of the guidelines (Allmänna Barnhuset, 1969). As an empirical matter, however, the matching procedures that were used continued to produce some positive correlations between the characteristics of adoptive and biological parents. Björklund et al. (2006) report modest positive correlations in income and education in a Swedish sample of adoptive and biological parents to children born between 1962 and 1966.

At the time of the adoption, the adoptive parents were provided with some information about the biological parents. Above all, according to new regulation in 1960 (1960 års Barnavårdslag (SFS 1960:97)), the adoptive parents were entitled to take part of the mandatory evaluation of the adoptee and the biological parents. This document included information about the names and some demographic and socioeconomic characteristics

of the biological parents such as age, educational attainment and any medical history. However, neither the evaluation report nor the decision contained any contact information for the biological parents such as addresses or personal identification number (Nordlöf, 2001).[2]

Still, it is relatively easy for an adoptee to find out who his or her biological parents are. The Swedish authorities keep detailed records of the biological mothers and (when known) fathers of all adoptees and adopted children have a legal right to request and obtain this information. Although the evidence is scant, such inquires seem to be relatively rare. Based on a thirteen percent sample of all 6,737 adoptions in Stockholm between 1918 and 1973, Nordlöf (2001) reports that 22 out of 866 (2.5%) adult adoptees had requested information about their biological parents from the official records. Moreover, interview studies with former social workers suggest that recurrent contacts between adoptees and their biological parents are extremely rare (Lindblom, 2001).

There were only few formal rules concerning who was allowed to adopt a child (Allmänna Barnhuset, 1969). Prospective parents were required to be at least 25 years old and it was rare for the adoptive mother's age to exceed 40. The justification for the upper age bound was that it should not be obvious from the adoptive mother's age alone that she was not the child's biological mother. The adoptive fathers were expected to have a stable source of income and adequate housing. More informally, the guidelines recommended that the social worker in charge of the adoption case should strive to find parents that were reasonably intelligent, tolerant, and empathetic.

There were also some important differences between the biological and adoptive parents (Bohman, 1970; Nordlöf, 2001; Björklund et al., 2006). The average biological mother of a child given up for adoption was typically young, unwed and likely to come from disadvantaged socioeconomic backgrounds. Adoptive parents, many of which resorted to adoption because of infertility, were naturally older. Compared to a representative sam-

[2]Personal communication with Barbro Nordl´of (2017-02-07) — a former social worker with responsibility for adoption cases and the author of one of the main sources for this section — corroborated the conclusion that the adoptive parents in general had no contact information about the biological parents.

ple of parents, the adoptive parents were more likely to come from upper socioeconomic strata. In all cases, however, there is substantial overlap in the distributions of characteristics between the adoptive and biological parents.

The evaluation literature has shown that children given up for adoption had lower birth-weights and were at slightly greater risk of congenital defects (Bohman, 1970; Nordlöf, 2001). At the same time, children perceived to have severe health problems or whose parents suffered from mental illness were seldom placed in adoptive homes but instead put into foster or institutional care. Bohman (1970) shows that the average health status of a sample of adoptees aged 10 to 11 was indistinguishable from a sample of non-adopted children. The lack of difference between the two groups probably masks two opposite forces of selection that roughly cancel out. In terms of health status and parental background, this suggests that adoptees were a positively selected subgroup from a slightly negatively selected pool of children.

Four important conclusions directly related to the identification assumptions discussed in the section on the empirical framework follow from this description of the Swedish adoption system. First, in a large majority of cases the children were placed with their adoptive parents early after birth, most often between 3 and 12 months of age. Second, despite the change in the guidelines to social workers on matching children to parents with similar cognitive and physical characteristics, it seems likely that some selective placement persisted. Third, the adoptive parents in general had only limited information about the biological parents and according to evaluation studies only a small fraction of adult adoptees requested and obtained further information about their birth parents from the official records. Fourth, there are differences both between (i) the adoptive and the own-birth children and (ii) the adoptive and the biological parents. These conclusions inform the set of sensitivity checks that we present in Appendix D.

Appendix C: Sample Restrictions and Summary Statistics

The following two tables contain additional information on sample restrictions (Table A1) and descriptive statistics (Table A2). As described in the main text, we construct our two samples using information from the Multi-Generation Registry (MGR) held by Statistics Sweden. Table A1 lists the sample restrictions we impose and the corresponding effects on the sample sizes. Our raw dataset contains 10,717,814 non-adopted and 155,865 individuals born between 1931 and 2014. We first restrict our samples to individuals having at least one identified household parent (biological parent in the non-adoptee sample and adoptive parent in the adoptee sample). Furthermore, both samples are restricted to children i) born between 1960 and 1980, ii) who were alive at the time of the first election for which they were eligible to vote and stand as a candidate and whose parents were alive by the time of the first election in 1982, and iii) who were reared by the same parents until age 16.

We impose a number of additional restrictions on the adoptee sample. First, to enable estimation of both pre- and post-birth factors, at least one biological parent needs to be identified in the official records. Second, to rule out the cases where a child was living with one biological parent (most often the biological mother) and adopted by the parent's spouse/partner (most often an adoptive father), we restrict the adoptee sample to children for whom two adoptive parents can be identified. Finally, we have dropped the few cases in which the child was adopted by a relative (grandparents, uncles and aunts).

Table A2 reports baseline probabilities of running for office for ownbirth and adopted children and their parents across the ten elections between 1982 and 2014. The entries also display the average parental and child age at the time of each election (in parentheses) and number of individuals (in brackets). Reflecting the fact that the children are very young at the beginning of the study period (the oldest children are 22 years of age at the time of the first election in 1982) whereas the parents on average are in their

seventies by 2014, the probability of standing as a candidate is increasing among children and decreasing among the parents over time. Another feature to be noted is the upward shift in the baseline probability between the elections in 1988 and 1991 across all groups. As discussed in the main text, this is due to the fact that we only have data on nominations to the municipality assemblies for the first three elections. For the remaining seven elections, the candidate information covers all three levels: the national parliament, the county level assemblies and the municipal level assemblies.

Table A1: Sample Restrictions

	Non-adoptees	Adoptees	Change in non-adoptees	Change in adoptees
All individuals in MGR	10,717,814	155,865		
Drop children with no household parents	8,984,409	149,432	-1,733,405	-6,433
Keep children born 1960-1980	2,424,821	49,579	-6,559,588	-99,853
Keep if the child and parent alive in 1982	2,385,127	49,293	-39,694	-286
Keep if the child was reared by the same parents	1,958,341	46,090	-426,786	-3,203
Keep if at least one biological parent is identified		28,896		-17,194
Keep if the child was adopted by two parents		10,434		-18,462
Drop if the child was adopted by relatives		10,141		-293

Table A2: Political Candidacy Across Elections

	1982	1985	1988	1991	1994	1998	2002	2006	2010	2014
Ownbirth Sample										
Children	0.30	0.35	0.36	0.47	0.55	0.51	0.54	0.65	0.72	0.80
	(20.1)	(21.5)	(23.1)	(24.7)	(26.4)	(28.7)	(32.6)	(36.6)	(40.6)	(44.6)
	[470,112]	[797,634]	[1,092,712]	[1,371,031]	[1,625,295]	[1,926,065]	[1,944,452]	[1,939,568]	[1,933,467]	[1,927,149]
Birth Fathers	1.95	2.02	1.85	2.14	1.90	1.66	1.52	1.39	1.29	1.15
	(43.1)	(46.0)	(48.8)	(51.7)	(54.5)	(58.1)	(61.7)	(65.2)	(68.6)	(72.0)
	[999,602]	[989,155]	[976,518]	[960,950]	[942,295]	[910,908]	[871,978]	[824,033]	[766,728]	[711,525]
Birth Mothers	0.92	0.98	1.01	1.24	1.21	1.03	0.91	0.81	0.75	0.66
	(40.1)	(43.1)	(46.0)	(49.0)	(51.9)	(55.8)	(59.6)	(63.4)	(67.0)	(70.6)
	[1,047,317]	[1,043,074]	[1,037,543]	[1,030,480]	[1,021,810]	[1,006,543]	[985,471]	[956,919]	[918,344]	[876,924]
Adoption Sample										
Children	0.35	0.41	0.44	0.60	0.77	0.67	0.80	0.97	1.04	1.01
	(20.1)	(21.9)	(24.0)	(26.4)	(28.9)	(32.2)	(36.2)	(40.2)	(44.2)	(48.2)
	[4,621]	[7,052]	[8,321]	[9,092]	[9,542]	[9,988]	[9,973]	[9,931]	[9,859]	[9,796]
Birth Fathers	0.64	0.53	0.48	0.72	0.72	0.77	0.76	0.85	0.92	0.85
	(44.7)	(47.4)	(50.0)	(52.5)	(55.1)	(58.6)	(61.9)	(65.2)	(68.4)	(71.5)
	[3,120]	[3,022]	[2,899]	[2,780]	[2,655]	[2,467]	[2,236]	[1,995]	[1,748]	[1,538]
Birth Mothers	0.37	0.46	0.44	0.70	0.76	0.76	0.50	0.41	0.63	0.68
	(40.5)	(43.4)	(46.3)	(49.2)	(52.1)	(55.9)	(59.6)	(63.2)	(66.8)	(70.3)
	[5,081]	[5,018]	[4,946]	[4,846]	[4,757]	[4,590]	[4,384]	[4,101]	[3,817]	[3,519]
Adoptive Fathers	3.16	3.29	2.84	2.92	2.33	2.19	1.76	1.64	1.09	1.14
	(51.6)	(54.5)	(57.4)	(60.2)	(63.0)	(66.6)	(70.1)	(73.5)	(76.6)	(79.7)
	[7,889]	[7,790]	[7,645]	[7,436]	[7,164]	[6,717]	[6,143]	[5,490]	[4,665]	[3,944]
Adoptive Mothers	1.38	1.41	1.34	1.63	1.36	1.21	0.97	0.79	0.69	0.47
	(49.1)	(52.0)	(55.0)	(57.9)	(60.8)	(64.7)	(68.4)	(72.1)	(75.5)	(78.9)
	[8,072]	[7,990]	[7,900]	[7,782]	[7,635]	[7,376]	[7,021]	[6,575]	[5,949]	[5,311]

Note: Probability of running for office in percentage points, average age at the time of each election (in parentheses), and number of individuals (in brackets) across election years for adopted and ownbirth children born 1960-1980 and their parents.

Appendix D: Auxiliary Analyses

The following eight tables contain additional analyses. Table A3 reports logit estimates corresponding to the linear probability estimates displayed in Table 2 in the main text. In order not to lose too many observations due to empty cells, we control for birth-cohort dummies instead of birth-year dummies in logit models. Comparing the results in Table 2 in the main text and Table A2, we can see that the findings are very similar if we use a logit estimator instead of OLS.

In our main analyses, we use a dummy indicator for standing as a candidate at least once in the ten elections between 1982 and 2014 as the primary outcome. Tables A4 and A5 provides transmission estimates in the ownbirth and adoption samples using two alternative outcomes: the number of times parents and children ran for office (Table A4) and being nominated at least twice (Table A5) during the study period. The pre-birth estimates are somewhat weaker in these models. This is most likely an effect of the very small amount of variation in birth parent candidacy status when using the more restrictive outcomes. However, the overall pattern of results is still similar to the one reported in Table 2 in the main text: irrespective of measure used i) there is a strong transmission from parent to child; ii) the transmission from mothers to children is somewhat stronger than the father-child association; and iii) the intergenerational transmission in candidacy status can be decomposed into both pre-birth and post-birth factors.

As noted in the main text, the bulk of the variation in the outcome measures is driven by candidates at the municipality level. In Tables A6 through A8 we report estimates from transmission models across the three levels of political office: municipal (Table A6), regional/county (Table A7) and national (Table A8). As should be expected, the main pattern of results is very similar to the one shown in Table 2 in the main text when using an indicator for running for the municipal office as the outcome.

The results are less consistent in Tables A7 and A8. On the one hand, the transmission rates for standing as a candidate at least once across the ten elections at the county and

national levels in the ownbirth sample are still very precisely estimated and substantively large, especially given the very low baseline probabilities among the children and parents: around 1% (running for county level office at least once) and 0.5% (running for the national parliament at least once). On the other hand, the pre- and post-birth effects are generally non-significant, in many cases negative, and in a few cases even significantly negative.

The most likely explanation for these results is the extremely low joint probability of encountering cases where both parent and child run for office at the regional and national levels. For example, in our adoption sample there is not a single dyad (biological father-child; biological mother-child; adoptive father-child; adoptive mother-child) in which both parent and child have run for the national parliament. It would take less than one such dyad to turn many of the negative effects in Table A8 positive. The corresponding figures for the county level outcome is a total of six dyads (five adoptive parent-child and one biological parent-child) in which both parent and child have run for office. These six cases are enough to produce positive point estimates for the pre-and post-birth effects in column 1 of Table A7. Put differently, to be well-powered enough to more precisely estimate the expected transmission rates for running for office at the county and national levels, we would need an adoption sample that is at least an order of magnitude larger than the one we currently have access to.

Another possible objection to our choice of outcome - running for office at least once across the ten elections between 1982 and 2014 — is that many of the candidates can be considered to be "listfillers" and thus unlikely to have any real political career. Against this backdrop, Tables A9 reports transmission estimates for winning office at least once. The results for winning office are very similar to the ones obtained for running at the county (Table A7) and national (Table A8) levels. Once again, we fail to obtain precise estimates for the pre- and post-birth effects in the adoption sample due to the very low baseline probabilities of getting elected. The chances of winning office at least once among the children in the sample is 0.8%. The corresponding figures for the four set of

parents are 0.5% (biological father), 2.6% (adoptive fathers), 0.4% (biological mothers and 1.3% (adoptive mothers). As for the joint occurrence of both parent and child winning office, our sample contains four such dyads (three adoptive parent-child and one biological parent-child). This, of course, means that the sign and magnitude of the estimates of the pre- and post-birth effects are very sensitive to small changes to the frequency of these cases. For example, if duplicating the only observation in which both the child and one of the biological parents ran for office the estimated pre-birth effect increases from 0.009 to 0.023. Likewise, if duplicating the three observations in which both the child and one of the adoptive parents ran for office the estimated post-birth effect increases from 0.003 to 0.012.

Two more things should be noted about the results in Table A9 and the fact that we focus on the indicator for candidacy in the main text. First, the fact that more than 98% of all the eligible voters are eliminated in the nomination stage reflects the vital role of candidate selection. Put simply, the real hurdle to clear is not so much getting elected conditional on being nominated, but instead getting one's name on the party list in the first place. Second, failure to get elected does not necessarily preclude individuals at the lower end of the party lists from reaching different political positions. Above all, non-elected candidates are commonly used to populate the many municipal boards and committees. In 2007 and 2011 Statistics Sweden conducted population surveys on all elected and non-elected representatives in Sweden's municipality and county councils. About 60% of the non-elected candidates in the 2006 and 2010 municipal and county elections were members of one or more board or committee in 2007 and 2011. Thus, viewing candidates with less electable list positions as "listfillers" may not be entirely correct.

Nevertheless, to provide a test for the degree to which our main results are driven by candidates lower or higher on the list Table A10 presents results for the transmission in standing as a top candidate, defined as being placed on the upper half of the party list. Even after eliminating many of the potential "listfillers," the estimates in Table A10 are

very similar to the results presented in Table 2 in the main text.

Table A3: Logit – Coefficient Estimates and Marginal Effects

	1	2	3	4	5	6
Ownbirth Sample						
Birth Parent	1.343***	–	–	–	–	–
	(0.013)	–	–	–	–	–
	[0.051]	–	–	–	–	–
Birth Father	–	1.265***	–	1.266***	–	1.041***
	–	(0.015)	–	(0.015)	–	(0.016)
	–	[0.049]	–	[0.049]	–	[0.036]
Birth Mother	–	–	1.380***	–	1.379***	1.083***
	–	–	(0.016)	–	(0.017)	(0.018)
	–	–	[0.058]	–	[0.058]	[0.039]
N	1,694,804	1,635,951	1,674,697	1,591,302	1,591,302	1,591,302
Adoption Sample						
Birth Parent	0.784***	–	–	–	–	–
	(0.223)	–	–	–	–	–
	[0.036]	–	–	–	–	–
Birth Father	–	1.138***	–	1.088***	–	1.009***
	–	(0.312)	–	(0.340)	–	(0.367)
	–	[0.060]	–	[0.057]	–	[0.049]
Birth Mother	–	–	0.357	–	0.191	−0.030
	–	–	(0.338)	–	(0.472)	(0.495)
	–	–	[0.014]	–	[0.007]	[−0.001]
Adoptive Parent	0.753***	–	–	–	–	–
	(0.162)	–	–	–	–	–
	[0.033]	–	–	–	–	–
Adoptive Father	–	0.635***	–	0.662***	–	0.476*
	–	(0.235)	–	(0.246)	–	(0.263)
	–	[0.027]	–	[0.028]	–	[0.018]
Adoptive Mother	–	–	1.139***	–	0.806***	0.653*
	–	–	(0.214)	–	(0.315)	(0.347)
	–	–	[0.059]	–	[0.038]	[0.027]
Prebirth+Postbirth	1.537***	1.773***	1.496***	1.750***	0.997*	1.485***/0.623
	(0.272)	(0.377)	(0.386)	(0.404)	(0.552)	(0.428)/(0.586)
	[0.069]	[0.087]	[0.073]	[0.085]	[0.038]	[0.067]/[0.026]
N	8,756	5,114	8,352	4,510	4,511	4,510

Note: Logit regression. The upper entries display coefficient estimates. The middle entries (in parentheses) display standard errors clustered by parent in the upper panel and by adoptive parent in the lower panel. The lower entries [in brackets] display marginal effects of discrete changes in parental candidacy status on the probability of running for office over the response surface. All models include controls for child's gender, 7 child birth-cohort dummies, 8 parental birth-cohort dummies, and 24 dummies for parents' county of residency. ***/**/*, indicates significance at the 1/5/10% level.

Table A4: Transmission Coefficients for Political Candidacy – Number of Nominations

	1	2	3	4	5	6
Ownbirth Sample						
Birth Parent	0.037***	–	–	–	–	–
	(0.001)	–	–	–	–	–
Birth Father	–	0.036***	–	0.036***	–	0.031***
	–	(0.001)	–	(0.001)	–	(0.001)
Birth Mother	–	–	0.043***	–	0.043***	0.035***
	–	–	(0.001)	–	(0.001)	(0.001)
N	1,694,804	1,635,951	1,674,697	1,591,302	1,591,302	1,591,302
Adoption Sample						
Birth Parent	0.018*	–	–	–	–	–
	(0.010)	–	–	–	–	–
Birth Father	–	0.029	–	0.027	–	0.026
	–	(0.021)	–	(0.021)	–	(0.022)
Birth Mother	–	–	0.007	–	−0.005	−0.007
	–	–	(0.011)	–	(0.009)	(0.009)
Adoptive Parent	0.033***	–	–	–	–	–
	(0.009)	–	–	–	–	–
Adoptive Father	–	0.032**	–	0.037**	–	0.030**
	–	(0.014)	–	(0.015)	–	(0.014)
Adoptive Mother	–	–	0.054***	–	0.050**	0.044**
	–	–	(0.018)	–	(0.024)	(0.022)
Prebirth+Postbirth	0.050***	0.061**	0.061***	0.064**	0.045*	0.056**/0.037
	(0.014)	(0.024)	(0.021)	(0.026)	(0.023)	(0.025)/(0.024)
N	8,756	5,145	8,352	4,538	4,538	4,538

Note: Linear regression. The columns display results using an indicator for the number of times the children ran for office across the ten elections between 1982 and 2014 as outcome. In the upper panel the standard errors (in parentheses) are clustered by parent. In the lower panel the standard errors are clustered by adoptive parent. All models include controls for child's gender, child birth-year dummies, parents birth-year dummies, and 24 dummies for parents' county of residency. ***/**/*, indicates significance at the 1/5/10% level.

Table A5: Transmission Coefficients for Political Candidacy – Nominated Twice

	1	2	3	4	5	6
Ownbirth Sample						
Birth Parent	0.036***	–	–	–	–	–
	(0.001)	–	–	–	–	–
Birth Father	–	0.036***	–	0.036***	–	0.032***
	–	(0.001)	–	(0.001)	–	(0.001)
Birth Mother	–	–	0.041***	–	0.041***	0.034***
	–	–	(0.001)	–	(0.001)	(0.001)
N	1,694,804	1,635,951	1,674,697	1,591,302	1,591,302	1,591,302
Adoption Sample						
Birth Parent	0.020	–	–	–	–	–
	(0.014)	–	–	–	–	–
Birth Father	–	0.022	–	0.014	–	0.015
	–	(0.021)	–	(0.020)	–	(0.020)
Birth Mother	–	–	0.016	–	−0.015***	−0.014**
	–	–	(0.017)	–	(0.004)	(0.006)
Adoptive Parent	0.020**	–	–	–	–	–
	(0.008)	–	–	–	–	–
Adoptive Father	–	0.028**	–	0.036**	–	0.031**
	–	(0.013)	–	(0.014)	–	(0.014)
Adoptive Mother	–	–	0.034**	–	0.035	0.026
	–	–	(0.017)	–	(0.022)	(0.021)
Prebirth+Postbirth	0.041***	0.050**	0.050**	0.049**	0.020	0.045*/0.012
	(0.016)	(0.024)	(0.023)	(0.024)	(0.022)	(0.024)/(0.022)
N	8,756	5,145	8,352	4,538	4,538	4,538

Note: Linear regression. The columns display results using an indicator for running for office at least twice across the ten elections between 1982 and 2014 as outcome. In the upper panel the standard errors (in parentheses) are clustered by parent. In the lower panel the standard errors are clustered by adoptive parent. All models include controls for child's gender, child birth-year dummies, parents birth-year dummies, and 24 dummies for parents' county of residency. ***/**/*, indicates significance at the 1/5/10% level.

Table A6: Transmission Coefficients for Political Candidacy – Municipal Assembly

	1	2	3	4	5	6
Ownbirth Sample						
Birth Parent	0.053***	–	–	–	–	–
	(0.001)	–	–	–	–	–
Birth Father	–	0.051***	–	0.051***	–	0.044***
	–	(0.001)	–	(0.001)	–	(0.001)
Birth Mother	–	–	0.061***	–	0.061***	0.051***
	–	–	(0.001)	–	(0.001)	(0.001)
N	1,694,804	1,635,951	1,674,697	1,591,302	1,591,302	1,591,302
Adoption Sample						
Birth Parent	0.033**3	–	–	–	–	–
	(0.014)	–	–	–	–	–
Birth Father	–	0.053**	–	0.048**	–	0.045*
	–	(0.023)	–	(0.024)	–	(0.024)
Birth Mother	–	–	0.013	–	0.006	−0.001
	–	–	(0.015)	–	(0.018)	(0.017)
Adoptive Parent	0.032***	–	–	–	–	–
	(0.009)	–	–	–	–	–
Adoptive Father	–	0.024*	–	0.032***	–	0.026*
	–	(0.013)	–	(0.014)	–	(0.014)
Adoptive Mother	–	–	0.055***	–	0.040*	0.034
	–	–	(0.016)	–	(0.021)	(0.022)
Prebirth+Postbirth	0.064***	0.077***	0.068***	0.080***	0.046*	0.071***/0.033
	(0.016)	(0.025)	(0.021)	(0.027)	(0.027)	(0.026)/(0.027)
N	8,756	5,145	8,352	4,538	4,538	4,538

Note: Linear regression. The columns display results using an indicator for running for the municipal assembly at least once across the ten elections between 1982 and 2014 as outcome. In the upper panel the standard errors (in parentheses) are clustered by parent. In the lower panel the standard errors are clustered by adoptive parent. All models include controls for child's gender, child birth-year dummies, parents birth-year dummies, and 24 dummies for parents' county of residency. ***/**/*, indicates significance at the 1/5/10% level.

Table A7: Transmission Coefficients for Political Candidacy – County Assembly

	1	2	3	4	5	6
Ownbirth Sample						
Birth Parent	0.024***	–	–	–	–	–
	(0.001)	–	–	–	–	–
Birth Father	–	0.026***	–	0.026***	–	0.024***
	–	(0.001)	–	(0.001)	–	(0.001)
Birth Mother	–	–	0.025***	–	0.025***	0.023***
	–	–	(0.001)	–	(0.001)	(0.001)
N	1,694,804	1,635,951	1,674,697	1,591,302	1,591,302	1,591,302
Adoption Sample						
Birth Parent	0.006	–	–	–	–	–
	(0.013)	–	–	–	–	–
Birth Father	–	−0.006	–	−0.007	–	−0.002
	–	(0.005)	–	(0.005)	–	(0.008)
Birth Mother	–	–	0.008	–	−0.012**	−0.014**
	–	–	(0.017)	–	(0.005)	(0.005)
Adoptive Parent	0.019*	–	–	–	–	–
	(0.011)	–	–	–	–	–
Adoptive Father	–	0.021	–	0.025	–	0.024
	–	(0.018)	–	(0.020)	–	(0.021)
Adoptive Mother	–	–	0.015	–	0.009	0.009
	–	–	(0.016)	–	(0.019)	(0.019)
Prebirth+Postbirth	0.025	0.015	0.023	0.018	−0.002	0.022/−0.004
	(0.017)	(0.018)	(0.023)	(0.020)	(0.019)	(0.022)/(0.020)
N	8,756	5,145	8,352	4,538	4,538	4,538

Note: Linear regression. The columns display results using an indicator for running for the county assembly at least once across the ten elections between 1982 and 2014 as outcome. In the upper panel the standard errors (in parentheses) are clustered by parent. In the lower panel the standard errors are clustered by adoptive parent. All models include controls for child's gender, child birth-year dummies, parents birth-year dummies, and 24 dummies for parents' county of residency. ***/**/*, indicates significance at the 1/5/10% level.

Table A8: Transmission Coefficients for Political Candidacy – National Parliament

	1	2	3	4	5	6
Ownbirth Sample						
Birth Parent	0.026***	–	–	–	–	–
	(0.002)	–	–	–	–	–
Birth Father	–	0.027***	–	0.027***	–	0.025***
	–	(0.002)	–	(0.002)	–	(0.002)
Birth Mother	–	–	0.028***	–	0.027***	0.026***
	–	–	(0.002)	–	(0.002)	(0.002)
N	1,694,804	1,635,951	1,674,697	1,591,302	1,591,302	1,591,302
Adoption Sample						
Birth Parent	−0.002	–	–	–	–	–
	(0.002)	–	–	–	–	–
Birth Father	–	−0.004	–	−0.005	–	−0.007
	–	(0.004)	–	(0.005)	–	(0.009)
Birth Mother	–	–	−0.005**	–	−0.016***	−0.014*
	–	–	(0.002)	–	(0.006)	(0.008)
Adoptive Parent	−0.003*	–	–	–	–	–
	(0.002)	–	–	–	–	–
Adoptive Father	–	−0.004	–	−0.003	–	−0.002
	–	(0.004)	–	(0.005)	–	(0.007)
Adoptive Mother	–	–	−0.003	–	−0.004	−0.007
	–	–	(0.002)	–	(0.004)	(0.005)
Prebirth+Postbirth	−0.005**	−0.008	−0.008**	−0.008	−0.020**	−0.008/−0.020*
	(0.003)	(0.006)	(0.004)	(0.006)	(0.008)	(0.010)/(0.011)
N	8,756	5,145	8,352	4,538	4,538	4,538

Note: Linear regression. The columns display results using an indicator for running for the national parliament at least once across the ten elections between 1982 and 2014 as outcome. In the upper panel the standard errors (in parentheses) are clustered by parent. In the lower panel the standard errors are clustered by adoptive parent. All models include controls for child's gender, child birth-year dummies, parents birth-year dummies, and 24 dummies for parents' county of residency. ***/**/*, indicates significance at the 1/5/10% level.

Table A9: Transmission Coefficients for Political Candidacy – Elected Candidates

	1	2	3	4	5	6
Ownbirth Sample						
Birth Parent	0.020***	–	–	–	–	–
	(0.001)	–	–	–	–	–
Birth Father	–	0.021***	–	0.021***	–	0.020***
	–	(0.001)	–	(0.001)	–	(0.001)
Birth Mother	–	–	0.021***	–	0.021***	0.019***
	–	–	(0.001)	–	(0.001)	(0.001)
N	1,694,804	1,635,951	1,674,697	1,591,302	1,591,302	1,591,302
Adoption Sample						
Birth Parent	0.009	–	–	–	–	–
	(0.015)	–	–	–	–	–
Birth Father	–	0.031	–	0.034	–	0.036
	–	(0.040)	–	(0.043)	–	(0.044)
Birth Mother	–	–	−0.006**	–	−0.007*	−0.007
	–	–	(0.003)	–	(0.004)	(0.006)
Adoptive Parent	0.003	–	–	–	–	–
	(0.006)	–	–	–	–	–
Adoptive Father	–	0.016	–	0.018	–	0.019
	–	(0.012)	–	(0.014)	–	(0.014)
Adoptive Mother	–	–	−0.008***	–	−0.005	−0.006
	–	–	(0.003)	–	(0.004)	(0.005)
Prebirth+Postbirth	0.012	0.047	−0.014***	0.052	−0.012**	0.055/−0.013
	(0.016)	(0.042)	(0.004)	(0.046)	(0.006)	(0.046)/(0.008)
N	8,756	5,145	8,352	4,538	4,538	4,538

Note: Linear regression. The columns display results using an indicator for winning elected at least once across the ten elections between 1982 and 2014 as outcome. In the upper panel the standard errors (in parentheses) are clustered by parent. In the lower panel the standard errors are clustered by adoptive parent. All models include controls for child's gender, child birth-year dummies, parents birth-year dummies, and 24 dummies for parents' county of residency. ***/**/*, indicates significance at the 1/5/10% level.

Table A10: Transmission Coefficients for Political Candidacy – Top Candidates

	1	2	3	4	5	6
Ownbirth Sample						
Birth Parent	0.045***	–	–	–	–	–
	(0.001)	–	–	–	–	–
Birth Father	–	0.046***	–	0.046***	–	0.041***
	–	(0.001)	–	(0.001)	–	(0.001)
Birth Mother	–	–	0.053***	–	0.052***	0.045***
	–	–	(0.001)	–	(0.001)	(0.001)
N	1,694,804	1,635,951	1,674,697	1,591,302	1,591,302	1,591,302
Adoption Sample						
Birth Parent	0.023*	–	–	–	–	–
	(0.013)	–	–	–	–	–
Birth Father	–	0.028	–	0.022	–	0.024
	–	(0.023)	–	(0.023)	–	(0.024)
Birth Mother	–	–	0.018	–	0.013	0.008
	–	–	(0.016)	–	(0.021)	(0.020)
Adoptive Parent	0.027***	–	–	–	–	–
	(0.009)	–	–	–	–	–
Adoptive Father	–	0.023*	–	0.029**	–	0.025*
	–	(0.012)	–	(0.013)	–	(0.013)
Adoptive Mother	–	–	0.039**	–	0.021	0.014
	–	–	(0.016)	–	(0.019)	(0.019)
Prebirth+Postbirth	0.049***	0.052**	0.057**	0.051*	0.034	0.049*/0.022
	(0.016)	(0.025)	(0.022)	(0.026)	(0.028)	(0.027)/(0.027)
N	8,756	5,145	8,352	4,538	4,538	4,538

Note: Linear regression. The columns display results using an indicator for being placed on the top half of the party list at least once across the ten elections between 1982 and 2014 as outcome. In the upper panel the standard errors (in parentheses) are clustered by parent. In the lower panel the standard errors are clustered by adoptive parent. All models include controls for child's gender, child birth-year dummies, parents birth-year dummies, and 24 dummies for parents' county of residency. ***/**/*, indicates significance at the 1/5/10% level.

Appendix E: Sensitivity Analyses

As noted above, the interpretation of the coefficients of biological parent candidacy status as pre-birth and adoptive parent candidacy status as post-birth factors rests on several important assumptions regarding the timing of adoption, random assignment, and the distributions from which children and parents are drawn. Here, we examine these assumptions and conduct sensitivity analyses to examine the robustness of our results to departures from them.

The first problem to consider is age at adoption. If adoptees live with their biological parents for a significant period of time before they are transferred to their adoptive home, the estimated pre-birth effect is also likely to capture the influence of the initial environment. However, the available evidence suggests that most children who were registered for adoption were placed in special nursery homes very shortly after birth (Nordlöf, 2001). Consequently, there are no compelling reasons to believe that possible post-birth effects that come from the adoptees' birth parents bias the estimated pre-birth effects.

On the other hand, 9 out of 10 adoptees spent some time in institutionalized care before placement and this may have impacted subsequent development in ways that influence the estimates of the post-birth effects. Bohman (1970, p. 25), in an evaluation of the adoption system, concludes that "material conditions and staff were of high standard" but also points out that there is some evidence that the institutional stay delayed development (Klackenberg, 1956).

Unfortunately, there is no register information available on age at adoption. However, it is possible to obtain an estimate of the share of adoptees under a certain age living with their adoptive parents using the quinquennial censuses. For example, Björklund et al. (2006) and Lindquist et al. (2015) show that out of the adoptees born 11 to 13 months before the 1960, 1965, and 1970 censuses, 75 to 80 percent belonged to the adoptive parents' household by the time of the census. We find similar results for adoptees born

a year prior to the 1975 (81 percent) and 1980 (78 percent) censuses. Since there is no reason to believe that we would obtain different results for non-census years, we can conclude that approximately 80 percent of the adoptees were living with their adoptive parents within a year of their birth date. These results suggest that departures from the adoption-at-birth assumption are relatively minor and therefore are unlikely to bias the pre-birth and post-birth estimates to any great extent.

Our baseline results in Table 2 also hinge on the assumption that adoptees are randomly assigned to families, at least conditional on observables. Violations of the assumption will generally upwardly bias the estimates of the pre-birth and post-birth effects. As a first check of the plausibility of this assumption, we can look at the tetrachoric correlations between the biological and adoptive parents' candidacy status: r=0.07, p-value=0.23 (biological father-adoptive father); r=0.04, p-value=0.54 (biological father-adoptive mother); r=-0.04, p-value=0.60 (biological mother-adoptive father); r=0.02, p-value=0.73 (biological mother-adoptive mother). The estimates are reassuring and suggest that nonrandom placement with respect to candidacy status does not seem to be an issue. The results presented in rows 1a through 1c of Table A11 further substantiate this claim. Here, we examine how the baseline results from Table 2 (row 1a, Table A11) change if one omits the indicator for standing as a candidate for the biological (row 1b, Table A11) or the adoptive parents (row 1c, Table A11).[3] Reflecting the relatively weak correlations between biological and adoptive parents' candidacy status, it is not surprising that the estimates are very similar to the baseline results. The only noticeable change is that the estimated effect of the biological father's candidacy status increases from 0.49 to 0.54 when excluding the adoptive parents.

The pre-birth and post-birth estimates may also be biased due to concordance among biological and adoptive parents on traits other than candidacy status. For instance, in

[3]In the robustness checks we focus on the two main specifications from Table 2: the joint parental model using an indicator for at least one parent standing as a candidate (column 1 in Table 2) and the model including both mother's and father's candidacy status (column 6 in Table 2). The entries in columns 1 and 2 in in each row of Table A11 correspond to the entries in column 1 in Table 2. Columns 3 to 6 in Table A11 correspond to column 6 in Table 2.

their sample of adoptees born between 1962 and 1966, Björklund et al. (2006) report that the adoptive mother-biological mother and the adoptive father-biological father correlations for years of schooling are equal to 0.14. We find correlations of similar size based on our sample.[4] Therefore, a second robustness test is to control for as much relevant information as possible about the biological and adoptive parents' characteristics. We report results from such models in rows 2b and 3b in Table A11. Apart from the baseline covariates (the child's gender, birth-year indicators for the child and each parent, 25 county indicators for where each parent lived at the time of the child's birth, and the *seats-to-voters* indicator), these models include measures of birth (row 2b) and adoptive (row 3b) parents' years of schooling and occupational status (an indicator for white collar employment). Rows 2a and 3a show the result from the baseline specification with samples restricted to adoptees for whom all biological parent (row 2a) or adoptive parent (row 3a) characteristics are non-missing.

If features of the post-birth environments are correlated with the pre-birth factors, the estimated effect of the latter should should decrease when controlling for the adoptive parents' characteristics. Likewise, if the post-birth effects are driven by correlations with biological parents' characteristics, we should expect the estimated effects of adoptive parents' candidacy status to fall when controlling for the pre-birth environment. Overall, the results in rows 2b and 3b show that the estimated pre-birth and post-birth effects hardly move at all when controlling for observable parental characteristics. Thus, we conclude that the baseline transmission estimates are robust to omitted variables.[5]

Another related source of bias is the possibility that the biological parents stayed in contact with their children after they were given up for adoption. The chance for this to happen is arguably greater if the adoptive parents are from the same geographical area as the biological parents. In line with this argument, we restricted the adoption sample to adoptees whose biological and adoptive parents lived in different counties at the time

[4]The biological father-adoptive father correlation for years of schooling is equal to 0.15; the biological father-adoptive mother correlation is equal to 0.13; the biological mother-adoptive father correlation is equal to 0.20; and the biological mother-adoptive mother correlation is equal to 0.18. All p-values<0.01.

[5]Including additional covariates such as parental income does not alter these results.

of the child's birth.[6] The transmission coefficients based on this restricted sample are presented in row 4. Due to the smaller sample size, the estimates are somewhat less precise. However, the point estimates are similar to the baseline results in row 1a and imply that pre- and post-birth factors contribute equally to the overall transmission in political candidacy status.

Direct contact between the adoptees and their biological parents is not necessary to invalidate the interpretation of α_1 from equation 2 in the main text as a pre-birth effect. Simply knowing who one's biological parents are and what they do may lead to upwardly biased pre-birth estimates. However, the previous discussion of adoptions in Sweden suggests that adoptive parents in general have limited information on the whereabouts of the adoptee's biological parents and that only a small minority of the adoptees make use of their legal right to obtain information about the biological parents from the official records.

Still, we cannot rule out the possibility that our pre-birth estimates are inflated due to the fact that at least some of the adoptees had information about their biological parents. An indirect way of checking the sensitivity of our results to this potential confounder is to restrict parental candidacy status to parents who were nominated but not elected for office. The logic behind this crude test is simple. It is reasonable to assume that adoptees are more likely to know if their birth parents currently occupy a political office, or have done so in the past, than they are to know if their biological parents have ever run for office without being elected. Therefore, if the pre-birth estimates are (partly) driven by adoptees knowing whether their biological parents are politically active or not, restricting parental candidacy status to indicate nominated but not elected candidates should lead to shrinking pre-birth estimates.

The results from such a model are presented in row 5. Comparing the estimates in row 5 to the corresponding baseline estimates in row 1a, it is comforting to note that

[6] During the study period, Sweden was divided into 24 counties of varying sizes: from the smallest covering around 3,000 square kilometers to the largest covering almost 100,000 square kilometers (more than a fifth of Sweden's total area).

the pre-birth effects are of similar magnitude across the two models. Above all, there is no tendency for the pre-birth estimates to decrease in size when restricting the parental candidacy indicators to include only unsuccessful candidates.

Next, we consider the assumption that adopted and ownbirth children and their parents are drawn from the same distributions of children and parents. From Table 1 in the main text, we know that adoptive parents are positively selected and adoptees' birth parents are negatively selected in relation to biological parents of children in the ownbirth sample. Above all, adoptive parents tend to be somewhat older, better educated, and more likely to be employed in the white collar sector compared to the adoptees' birth parents. In case these differences bias our estimated pre-birth and post-birth effects, β_1 (as obtained from the sample of ownbirth children) may not be equal to the sum of α_1 and α_2 (from the adoption sample). For instance, it may be that highly educated parents are more effective at transmitting behavioral patterns to their offspring or, conversely, that there are diminishing marginal returns to improvements in the post-birth environment.

We address this problem by conducting two robustness checks, both of which involve reweighting the adoption sample to make it more comparable to the ownbirth sample. First, we reweight the adoption sample such that birth parents of the adoptees become more similar to the parents of the ownbirth children regarding educational attainment, occupational status, and age. The second reweighted sample is instead designed to make the adoptive parents more similar to the parents in the ownbirth sample.

To construct the set of weights, we first combine the ownbirth and adoption samples. The weights for the pre-birth environment are defined as the inverse of the propensity scores obtained from a logit model with an indicator for being adopted as outcome and biological mothers' and biological fathers' birth octiles, years of schooling, and occupational status in 1990 (white collar or not) as covariates. The procedure for constructing the weights based on the post-birth characteristics is analogous and uses rearing parents'

birth octiles, years of schooling, and occupational status as covariates.[7]

After reweighting the adoption sample, we estimate the baseline models using weighted least squares. In row 6b of Table A11 we report estimates from the models in which the adoption sample is reweighted such that the birth parents of the adoptees are more similar to the parents of the ownbirth children. Row 7b instead shows estimates from a sample that is reweighted to make the adoptive parents more similar to the parents in the ownbirth sample. Rows 6a and 7a display results from corresponding unweighted regression models with the sample restrictions implied by the weights. It should be noted that the aim of this procedure is not to perfectly equate the pre- and post-birth environments but, more modestly, to examine whether making the distribution of parental characteristics in the adoption sample more similar to the distribution of parental characteristics in the ownbirth sample influences the estimated pre- and post-birth effects.

Still, it is interesting that the estimates from the weighted regressions tend to be slightly smaller compared to those obtained in the corresponding unweighted models. For example, the unweighted effect of having at least one biological parent who ran for office according to the estimates in row 6a is 0.038 whereas the corresponding estimate from the weighted regression is equal to 0.026. Likewise, the post-birth effect from the weighted model (0.029 in row 7b) is smaller than the unweighted estimate (0.034 in row 7a). These results suggest that the positive difference between the sum of pre- and post-birth effects reported in the bottom row in Table 2 and the corresponding transmission estimate in the upper panel may be due to the fact that the estimates obtained in the adoption sample are somewhat inflated. However, it is important not to overemphasize the differences between the unweighted and weighted estimates. First, the estimates from the unweighted and weighted regressions are of similar magnitude and in the same direction. Second, in one case — the effect of the adoptive father — the weighted estimate (row 7b: 0.32) is actually larger in size compared to the corresponding unweighted coefficient (row 7a: 0.23).

[7] Concretely, this means that adopted children with a higher likelihood of *not* being adopted given the covariates (that is, observations with larger inverse propensity scores) are treated as more informative about the underlying relationship and therefore are given more weight in the regressions.

Table A11: Sensitivity Analyses

	Parent		Father		Mother	
	Birth	Adoptive	Birth	Adoptive	Birth	Adoptive
Nonrandom Placement: Exclude Info						
(1a) Basline results	0.035***	0.034***	0.049**	0.028**	0.004	0.034
(N=8,756/4,538)	(0.013)	(0.009)	(0.024)	(0.014)	(0.019)	(0.022)
(1b) Exclude info on birth parent	–	0.034***	–	0.025*	–	0.038*
(N=8,756/4,538)	–	(0.009)	–	(0.014)	–	(0.022)
(1c) Exclude info on adoptive parent	0.035***	–	0.054**	–	0.002	–
(N=8,756/4,538)	(0.013)	–	(0.024)	–	(0.019)	–
Nonrandom Placement: Include Info						
(2a) Basline results	0.038***	0.040***	0.052*	0.032*	−0.021	0.067**
(N=6,709/2,701)	(0.014)	(0.011)	(0.029)	(0.017)	(0.015)	(0.031)
(2b) Include info on birth parent	0.036***	0.040***	0.049*	0.032*	−0.024	0.066***
(N=6,709/2,701)	(0.014)	(0.011)	(0.029)	(0.017)	(0.016)	(0.031)
(3a) Basline results	0.036***	0.034***	0.061**	0.023	0.007	0.032
(N=8,262/3,727)	(0.013)	(0.009)	(0.028)	(0.014)	(0.020)	(0.023)
(3b) Include info on adoptive parent	0.036***	0.034***	0.062**	0.022	0.006	0.031
(N=8,262/3,727)	(0.013)	(0.009)	(0.027)	(0.014)	(0.020)	(0.023)
Birth and adoptive parents living in different counties						
(4) Living in different counties	0.051**	0.037***	0.023	0.039	0.016	0.055
(N=4,016/1,705)	(0.021)	(0.014)	(0.037)	(0.024)	(0.029)	(0.041)
Nominated but not elected parents						
(5) Nominated only	0.036**	0.022**	0.060**	0.027*	−0.001	0.029
(N=8,756/4,538)	(0.015)	(0.010)	(0.027)	(0.016)	(0.020)	(0.027)
Reweighted Samples						
(7a) Basline results	0.038***	0.040***	0.052*	0.032*	−0.021	0.067**
(N=6,709/2,701)	(0.014)	(0.011)	(0.029)	(0.017)	(0.015)	(0.031)
(7b) Reweight prebirth factors	0.026**	0.041***	0.038	0.038**	−0.023	0.055*
(N=6,709/2,701)	(0.013)	(0.013)	(0.029)	(0.017)	(0.016)	(0.030)
(8a) Baseline results	0.036***	0.034***	0.061**	0.023	0.007	0.032
(N=8,262/3,727)	(0.013)	(0.009)	(0.028)	(0.014)	(0.020)	(0.023)
(8b) Reweight postbirth factors	0.037**	0.029**	0.090**	0.032*	−0.008	0.020
(N=8,262/3,727)	(0.016)	(0.013)	(0.041)	(0.018)	(0.022)	(0.024)

Note: Linear regression. In the upper panel the standard errors (in parentheses) are clustered by parent. In the lower panel the standard errors are clustered by adoptive parent. All models include controls for child's gender, child birth-year dummies, parents birth-year dummies, and 24 dummies for parents' county of residency. ***/**/*, indicates significance at the 1/5/10 percent level.

Appendix F: External Validity

The main conclusion of the sensitivity checks discussed above is that the baseline estimates from Table 2 are reasonably robust to violations of the assumptions about random assignment and the distributions from which children and parents are drawn. However, although the estimates appear to be internally valid, a possible objection to our results is that they may not generalize to other countries and contexts. Sweden's electorate and political institutions stand out along many dimensions in cross-country comparisons. Particularly striking features include the high Swedish turnout rates (Birch, 2010) and considerable descriptive gender equality in representation (Besley et al., 2013). Considering the fact that all political behavior is embedded in specific institutional environments, it is fair to ask to what degree, if at all, the parameter estimates obtained in our study translate to other contexts. Unfortunately, our ability to directly answer this question is limited. One of the strengths of this study — the unique data — is also a weakness in this respect. We are not aware of any other country for which i) population data on political candidates is available and ii) the administrative registers allow us to identify parent-child pairs and adoption status.

To explore the external validity of our results we instead, as a second best alternative, compare parent-child transmission in three political traits related to candidacy status in a Swedish and a U.S. sample: voter turnout, political interest, and an indicator for having attended a party meeting or political rally. The U.S. models are based on the 1997 wave of the "Three Generations Combined, 1965–1997 (ICPSR 4532)" study (Elliot, 2006). To obtain Swedish samples with intergenerational data on political traits, we use information from two datasets: the turnout surveys carried out by Statistics Sweden in connection to each of the six general election between 1991 and 2010 and the yearly Living Conditions Surveys (ULF/SILC) also carried out by Statistics Sweden between 1980 and 2010. Even though there are no intergenerational components in the two Swedish surveys, the large sample sizes obtained when pooling all separate cross-sections give rise to a fair amount

of parent-child pairs with information on turnout, political interest, and party meeting attendance.

The parents in the U.S. sample were all born in 1947, and both parents and their children were surveyed in 1997. In order to minimize differences between the Swedish and U.S. samples and at the same time obtain reasonably large sample sizes, we restrict the Swedish sample to children whose parents were born between 1944 and 1950. Furthermore, we control for a similar set of baseline covariates in the transmission analyses. Details on sample construction, item wordings, and model specification are provided in Appendix A.

The results are presented in Table A12. To substantiate the claim that these traits are related to the tendency to run for office, the first column displays correlation coefficients between candidacy status and turnout, political interest, and the indicator for attending a party meeting or political rally for a sample of Swedes born between 1935 and 1970.[8] The correlations for turnout and political interest are moderate whereas the relationship between candidacy experience and the indicator for attending party meetings or political rallies is strong. Under the assumption that these correlations are as strong in the U.S. context as well, comparable transmission rates across the two countries would suggest that Sweden is not a unique case regarding the intergenerational association in candidacy status. In line with this argument, the transmission coefficients from the U.S. sample in column 3 are very similar to the corresponding Swedish estimates in column 2.

Another issue concerns the relative importance of the pre-birth and post-birth influences reported in this study. Once again, a proper examination of this question would require intergenerational data with information on adoption status and both adoptive and birth parents across several countries. However, related research on the heritability of political traits based on the classical twin design provides no evidence that behavior genetic parameters obtained from Swedish data vary systematically from those obtained from other countries. For example, reported heritability estimates for political participa-

[8]We use the tetrachoric correlation coefficient for the dichotomous traits (turnout and meeting attendance) and the polychoric correlation for the four-category measure of political interest.

tion are very similar in Sweden, Denmark, and the U.S. (Klemmensen et al., 2012; Dawes et al., 2014). Furthermore, a recent comparative study of the heritability of political orientations in Australia, Denmark, Hungary, Sweden, and the U.S. found only small differences across countries (Hatemi et al., 2014).[9]

Table A12: External Validity: Transmission in Other Samples

	Correlation to being Nominated	Transmission Sweden	Transmission U.S.
Turnout	0.272***	0.147***	0.141*
(N=271,755/5,949/581)	(0.007)	(0.016)	(0.073)
Political Interest	0.297***	0.212***	0.234***
(N=67,184/344/708)	(0.011)	(0.063)	(0.047)
Attended Political Meeting	0.687***	0.229**	0.166***
(N=60,200/243/709)	(0.009)	(0.114)	(0.037)

Note: Column one reports correlation coefficients (polychoric in row 1 and tetrachoric in rows 2 and 3) between the indicator for being nominated at least once during the six elections 1991–2010 and three measures of political participation in the Swedish sample. Columns 2 and 3 display ownbirth transmission coefficients in Swedish and U.S. samples. Standard errors (in parentheses) are clustered by parent. Controls for child's gender, parental age, child's age, and region of residency are included in all transmission models. Racial self-classification is also controlled for in the U.S. sample. ***/**/* indicates significance at the 1/5/10 percent level.

[9]A certain amount of caution is needed when discussing the pre-birth and post-birth effects in terms of genetic versus environmental effects. Above all, the impact of the birth mother in the adoption sample reflects both genetic effects and the influence of the uterine environment whereas genetic factors alone cause any influence of the birth father. However, since it is reasonable to assume that the genetic endowments and the prenatal environment are positively related and since we find no evidence that the maternal pre-birth effects are stronger than the paternal pre-birth influence, we believe that any prenatal effects on the probability of standing as a candidate are small in magnitude.

References

Allmänna Barnhuset (1955). *Adoption*. Stockholm: Direktionen över Allmänna Barnhuset i samråd med Medicinstyrelsen och Socialstyrelsen.

Allmänna Barnhuset (1969). *Adoption*. Stockholm: Direktionen över Allmänna Barnhuset i samråd med Medicinstyrelsen och Socialstyrelsen.

Besley, T., O. Folke, T. Persson, and J. Rickne (2013). *Gender quotas and the crisis of the mediocre man*. IFN Working Paper, No. 985, Research Institute of Industrial Economics (IFN), Stockholm.

Birch, S. (2010). Perceptions of electoral fairness and voter turnout. *Comparative Political Studies 43*(12), 1601–1622.

Björklund, A., M. Lindahl, and E. Plug (2006). The origins of intergenerational associations. *The Quarterly Journal of Economics 121*(3), pp. 999–1028.

Bohman, M. (1970). *Adopted children and their families*. Stockholm: Proprius.

Cesarini, D., M. Johannesson, and S. Oskarsson (2014). Pre-birth factors, post-birth factors, and voting. *American Political Science Review 108*(1), 71–87.

Dawes, C., D. Cesarini, J. H. Fowler, M. Johannesson, P. K. Magnusson, and S. Oskarsson (2014). The relationship between genes, psychological traits, and political participation. *American Journal of Political Science 58*(4), 888–903.

Elliot, P. (2006). Three generations combined, 1965–1997. ICPSR04532-v1. Chapel Hill, NC: University of North Carolina [producer]. Inter-university Consortium for Political and Social Research [distributor], 2007-03-23. doi:10.3886/ICPSR04532.v1.

Hatemi, P., R. Klemmensen, S. E. Medland, S. Oskarsson, L. Littvay, C. Dawes, B. Verhulst, R. McDermott, A. S. Nørgaard, C. Klofstad, et al. (2014). Genetic influences on political ideologies. *Behavior Genetics 44*(3), 282–294.

Hjalmarsson, R. and M. J. Lindquist (2013). The origins of intergenerational associations in crime. *Labour Economics 20*, 68–81.

Klackenberg, G. (1956). Studies in maternal deprivation in infants' homes. *Acta Paediatrica 45*(1), 1–12.

Klemmensen, R., P. K. Hatemi, S. B. Hobolt, I. Petersen, A. Skytthe, and A. S. Nørgaard (2012). The genetics of political participation, civic duty, and political efficacy across cultures. *Journal of Theoretical Politics 24*(3), 409–427.

Lindblom, A.-C. (2001). *Ds 2001:53: Föräldrars samtycke till adoption m.m.* Departementsserien.

Lindquist, M. J., J. Sol, and M. Van Praag (2015). Why do entrepreneurial parents have entrepreneurial children? *Journal of Labor Economics*.

Nordlöf, B. (2001). *Svenska adoptioner i Stockholm 1918-1973.* FoU-rapport 2001:8, Socialtjänstförvaltningen, Forsknings- och utvecklingsenheten.
